# Documented but Not Demonstrably Performed: A Retrospective Audit of Routine NRS-2002 Screening Records in Hospitalized Adults with Diabetes

**DOI:** 10.3390/healthcare14203527

**Published:** 2026-10-20

**Authors:** Nawaf W. Alruwaili, Ali M. Alnughaimish, Khaled H. Aburisheh, Abdulmajeed Altamimi, Abdulaziz Alkhathaami, Aljazi Bin Zarah, Nora Alafif

**Affiliations:** 1 Department of Community Health Sciences, College of Applied Medical Sciences, King Saud University, Riyadh 11433, Saudi Arabia; anughaimish@ksu.edu.sa (A.M.A.); abinzaraah@ksu.edu.sa (A.B.Z.); nalafeef@ksu.edu.sa (N.A.); 2 Clinical Nutrition Department, King Khalid University Hospital, King Saud University Medical City, King Saud University, Riyadh 12372, Saudi Arabia; aaltamimi3@ksu.edu.sa; 3 University Diabetes Center, King Saud University Medical City, King Saud University, Riyadh 11472, Saudi Arabia; kaburisheh@ksu.edu.sa; 4 Clinical Nutrition Department, King Abdulaziz University Hospital, King Saud University Medical City, King Saud University, Riyadh 12629, Saudi Arabia; aalkhathaami@ksu.edu.sa

**Keywords:** NRS-2002, nutritional-risk screening, diabetes mellitus, hospitalized patients, screening completeness, implementation fidelity, documentation quality, clinical audit, trained nurses

## Abstract

**Background/Objectives:** Nutritional-risk screening at admission is mandatory in many hospitals but is rarely audited. We audited routine Nutritional-Risk Screening 2002 (NRS-2002) records entered by nurses with mandatory training and credentialed access in hospitalized adults with diabetes. **Methods:** Retrospective, records-based audit of all eligible admissions (2020–2025) at a Saudi tertiary center. Routine items were extracted verbatim, and dietitians re-scored each admission from the same record using a common written procedure. **Results:** Of 650 admissions assessed, 131 were excluded (46 with no form opened; 85 with incomplete clinical data). Of 519 analyzed forms, 446 (85.9%) carried zero on every item and 22 (4.2%) contained any substantive item; of 73 non-zero totals, 41 comprised the age item alone and 11 could not be derived from their items. Among 83 admissions in which a charted body-mass index below 20.5 kg/m^2^ made the full screen mandatory, 79 (95.2%) had no substantive item. Any entry was present in 70.8% of patients aged ≥70 years versus 5.9% of younger patients, although indications for the full screen were less frequent in older patients. Re-scoring classified 124 admissions (23.9%, 95% CI 20.4–27.7) at NRS-2002-defined risk versus one routinely; 123 (99.2%) were unrecognized. Scoring diabetes itself as disease severity 1 left this unchanged; an extreme lower-bound rule gave 57 (11.0%), none recognized. **Conclusions:** Routine documentation was strongly suggestive of incomplete NRS-2002 implementation despite mandatory training. Its causes cannot be determined from the records; workflow-embedded completion, automated referral, and routine audit warrant prospective evaluation.

## 1. Introduction

Diabetes is a chronic disease that occurs when the pancreas does not produce enough insulin or when the body cannot effectively use the insulin it produces, leading to elevated blood glucose levels [1]. The number of people living with diabetes worldwide has risen from 200 million in 1990 to 830 million in 2022 [1]. In Saudi Arabia, the age-standardized prevalence of diabetes among adults aged 20–79 years was estimated at 23.1% in 2024, equivalent to more than 5.3 million affected individuals [2].

Disease-related malnutrition in hospitalized adults is consistently associated with adverse clinical outcomes [3,4]. Nutritional risk at admission is independently associated with increased mortality, prolonged length of stay, and hospital readmission [5,6], and Saudi data confirm a substantial local burden [7] alongside documented variation in nutritional support practice [8]. The Global Leadership Initiative on Malnutrition (GLIM) framework specifies a two-step process in which a validated screening tool first identifies patients at risk, followed by a formal diagnostic assessment [9]. The European Society for Clinical Nutrition and Metabolism recommends early detection of nutritional risk as part of routine hospital care [10] and endorses the Nutritional Risk Screening 2002 (NRS-2002) for this purpose [11]. The NRS-2002 combines an impaired nutritional status component (body-mass index [BMI], recent weight loss, and reduced dietary intake), a disease severity component, and an additional point for age ≥70 years [12].

Among hospitalized patients, those with diabetes are particularly vulnerable. A recent systematic review and meta-analysis reported a pooled prevalence of malnutrition of 33% and a prevalence of nutritional risk of 44% in this population [13], and nutritional risk has been linked to prolonged hospital stay in type 2 diabetes [14]. Expert recommendations emphasize early identification of malnutrition in these patients [15], and higher NRS-2002 scores have been reported to be associated with elevated HbA1c, ketosis, and infection, and with lower BMI and prealbumin [16].

The value of any screening program, however, depends less on the instrument’s psychometric properties than on whether it is actually applied. Existing tools differ in validity, reliability, and generalizability [17,18,19], and no single tool performs consistently across all hospital populations [18,19]; but a tool that is not completed cannot perform at all. Prior work has reported low agreement between nursing staff and researchers using the NRS-2002 [20], and between nurses and dietitians in a tertiary center [21], and has repeatedly described incomplete documentation of the parameters required for screening [22,23,24]. Such findings are commonly attributed to insufficient training, and recent evidence is read as supporting that view: trained nurses in the VAL-NUT study screened effectively within a study protocol [25], while among trained nurses screening patients with cancer, agreement with a dietitian reference was only fair overall and varied markedly across the instrument’s items [26]. These reports are nonetheless framed in terms of inter-rater reliability, which presupposes that two raters independently applied the same instrument. That assumption is often untenable in routine care, where the prior question is whether the screen was performed at all—and where training may already be in place.

The present study was therefore designed as a records-based audit rather than a reliability study, in a setting where NRS-2002 training is a mandatory institutional competency and access to the electronic screening module is restricted to nurses who have completed it, so that a complete absence of training and lack of access are unlikely explanations for any shortfall. Our primary objective was to quantify the completeness and item-level content of routine, nurse-documented NRS-2002 records in hospitalized adults with diabetes. Our secondary objective was to quantify the classification consequences of any incompleteness by comparing routine records with a record-based, standardized dietitian re-scoring of the same admission data. Our third, exploratory objective was to describe the clinical and laboratory features of patients whom re-scoring classified as at NRS-2002-defined nutritional risk, deliberately excluding the variables that constitute the score itself. We did not set out to establish the prevalence of malnutrition, which requires a reference-standard diagnostic assessment that was not available in these records, nor to identify the causes of incomplete screening, which records cannot reveal.

## 2. Materials and Methods

### 2.1. Study Design and Setting

This was a retrospective, records-based audit of nutritional-risk screening conducted at King Saud University Medical City (KSUMC), Riyadh, Saudi Arabia, in adult patients with diabetes mellitus admitted between January 2020 and December 2025. All admissions of adults with diabetes during this period identified in the electronic medical record were assessed for eligibility; no sampling was applied. The study is reported in accordance with the STROBE statement for cross-sectional studies [27]. The design deliberately does not constitute an inter-rater reliability study. The two sources of NRS-2002 scores compared here were generated under different conditions: routine nurse documentation was produced during clinical care by staff who were unaware that their entries would later be examined, whereas dietitian scores were produced retrospectively for research from the same electronic record, without blinding to the routine entry. These are therefore not independent parallel ratings of the same patient, and no conclusion about the relative accuracy of nurses versus dietitians as raters can be, or is, drawn. What the comparison does support is an assessment of whether the screening instrument was completed in routine practice, and of the classification consequences when it was not.

Nursing staff at the study institution are trained in the NRS-2002 as part of mandatory institutional competency requirements, and access to the electronic admission screening module is granted only to nurses who have completed that training. Every routine score analyzed here was therefore recorded by a nurse who had completed that training and been credentialed to use the module. We had no access to individual training dates, refresher training, competency assessments, nurse identity, staffing or workload, so the quality, recency, and retention of training could not be assessed. Mandatory training makes a complete absence of training an unlikely explanation for the findings, but it does not exclude deficits in knowledge retention, practical competency, reinforcement or implementation support.

The study was conducted in accordance with the Declaration of Helsinki and approved by the Institutional Review Board, Health Sciences Colleges Research on Human Subjects, King Saud University (Reference No. 26/0202/IRB; Project No. R26-IRB-114; approved 29 March 2026). The requirement for individual informed consent was waived owing to the retrospective use of de-identified records. Data were extracted from the electronic medical record after approval had been granted. No study protocol or statistical analysis plan was registered, and the framing of the study as an audit of documentation completeness was adopted after initial inspection of the routine records; the analyses are therefore descriptive and exploratory, and no confirmatory hypothesis was tested.

### 2.2. Participants

Eligible patients were adults aged ≥18 years at admission with a documented diagnosis of diabetes mellitus in the electronic medical record, admitted to KSUMC during the study period, and with an admission NRS-2002 form present in the record. Patients were excluded if no NRS-2002 form had been opened at admission, or if the clinical data required for standardized re-scoring (notably height and weight for BMI) were unavailable. The second criterion means that data completeness in the analytic dataset is partly a consequence of selection.

Of 650 admission records assessed for eligibility, 131 were excluded: 85 because the clinical data required for standardized re-scoring were incomplete, and 46 because no baseline NRS-2002 form had been opened at admission. The final analytic sample comprised 519 admissions. An important consequence of the eligibility criteria should be noted at the outset: because an NRS-2002 form had to be present, the cohort is restricted to admissions at which the screening process was at least initiated. Completeness estimates reported here therefore describe the content of existing forms and are likely to be optimistic across all admissions. The routine form content of the 85 admissions excluded for incomplete clinical data is not available in the analysis dataset, so the implementation pathway across all 650 assessed admissions is reported with bounds (Section 3.2).

### 2.3. Data Collection and Variables

Data were extracted retrospectively from the electronic medical record. Variables comprised demographics (age, sex); clinical characteristics (type and duration of diabetes, length of hospital stay, hypertension, coronary heart disease, diabetic kidney disease, neuropathy, retinopathy); anthropometry (height, weight, and BMI recorded at admission); and laboratory values (HbA1c, serum albumin, bicarbonate, and C-reactive protein [CRP]). The analytic identifier is a study-specific code assigned to each admission; the link to the medical record number was not retained in the de-identified extract, so admissions belonging to the same patient could not be identified. Ward, nurse identity, shift, time and route of admission, and the calendar date of admission were not extracted.

Temporal definitions were as follows. Anthropometric measurements were those recorded at admission. Laboratory values were the first result available within 24 h of admission. Ketosis denoted biochemically confirmed ketosis or diabetic ketoacidosis documented at, or during, the index admission. Infection denoted a clinically documented infection in the six months preceding admission; this window applies to infection only and not to ketosis. Data completeness for every variable is reported in Table S1: all variables were complete for the 519 patients except CRP, which was available for 111 patients (21.4%).

### 2.4. The NRS-2002 and the Two Sources of Scores

The NRS-2002 comprises an initial screen and a final screen. The initial screen asks four questions (BMI < 20.5 kg/m^2^, weight loss within the last 3 months, reduced dietary intake in the last week, and severe illness); if any is answered affirmatively the final screen is completed, and if all are negative the screen is repeated weekly. In the final screen, an impaired-nutritional-status subscore (0–3) is assigned from BMI, recent unintentional weight loss, and reduced dietary intake, and a disease-severity subscore (0–3) from the acute clinical condition and its associated increase in nutritional requirements. One point is added for age ≥70 years, and a total ≥3 denotes nutritional risk [11,12].

Routine scores. Nurse-documented NRS-2002 entries were extracted verbatim from the admission screening form, preserving the two subscores and the recorded total separately. No routine entry was modified, imputed, or corrected. The extracted fields carry a numeric value for every item, so a score that was deliberately assigned as zero and an item that was never completed are not distinguishable from the extract alone. We therefore describe such records throughout as carrying a score of zero rather than as blank, and treat non-completion as an inference from the pattern of the data—set out in Section 3.2 and Section 4—rather than as an observation. The extract did not include the initial-screen responses, item-entry timestamps, or the audit trail of the electronic form, and we could not verify whether item fields are pre-populated with zero by default. The data do show that the recorded total is not computed automatically from the subscores, because 11 totals exceed the sum of their subscores and any applicable age point (Section 3.2).

Standardized re-scoring. Every admission was re-scored by a clinical dietitian from the same electronic record, using a common written procedure applied across the cohort; ‘standardized’ refers to this common procedure and does not imply a validated reference standard. Because the dietitians did not examine the patients, the re-score measures the nutritional information documented in the record rather than the patient’s nutritional status itself. The impaired-nutritional-status subscore was assigned from the admission BMI together with documented weight change and documented oral intake in the week preceding admission, following the published NRS-2002 categories (BMI < 18.5 kg/m^2^ with impaired general condition, weight loss >5% in one month, or intake <25% of requirement scoring 3; the corresponding moderate and mild categories scoring 2 and 1). Impaired general condition was judged by the dietitian from the documented clinical state at admission, a judgment that cannot be audited retrospectively. The disease-severity subscore was assigned from the documented admission diagnosis and acute clinical condition, following the operational definitions of Kondrup et al. [12] (score 1, a chronic disease with complications and a modest increase in requirements; score 2, a patient confined to bed by illness with a substantial increase; score 3, an illness of intensive-care severity). The procedure did not award a disease-severity point for the diagnosis of diabetes alone. In this cohort the most frequent qualifying condition was decompensated diabetes with ketosis or ketoacidosis, scored according to the clinical severity of the metabolic decompensation and the associated increase in nutritional requirements rather than from any laboratory threshold; the distribution of disease-severity subscores across documented conditions is given in Table S12. Because Kondrup’s table lists diabetes among the examples for a disease-severity score of one [12], and because no objective threshold separated the severity grades, the classification was repeated under alternative scoring rules (Section 2.7). Re-scoring was performed by three clinical dietitians. The 519 admissions were divided equally and at random between them (173 each), and each admission was re-scored by a single dietitian, whose judgment resolved any ambiguous case. The identity of the scoring dietitian was not recorded in the analysis dataset, so differences between dietitians could not be examined, and no admission was scored by more than one dietitian, so the inter-rater reliability of the re-scoring was not estimated. The dietitians were not blinded to the routine nursing entry.

The age adjustment was applied as follows: patients aged ≥70 years received one additional point when at least one substantive subscore was greater than zero, consistent with the NRS-2002 structure in which the final screen is reached only after a positive initial screen. This convention was followed for 64 of the 65 patients aged ≥70 years; in the remaining patient the age point was recorded with both substantive subscores at zero (total score 1, not at risk). Applying the age point unconditionally to all 65 patients aged ≥70 years changed no patient’s risk classification (Table S2), so this convention has no bearing on the results.

Laboratory variables (albumin, HbA1c, bicarbonate, CRP) are not components of the NRS-2002 and were not used, at any point, to assign or modify any NRS-2002 item. No cut-offs were applied to them. They were analyzed only as descriptive correlates of the standardized score.

Missing data were not imputed. Because the eligibility criteria required the data necessary for re-scoring, all 519 included admissions could be re-scored on every NRS-2002 item.

### 2.5. Data Quality and Integrity Checks

Before analysis, the dataset was subjected to a systematic integrity audit covering duplicate records, out-of-range values, logical consistency between related fields, and the internal arithmetic of both sets of NRS-2002 scores. Body-mass index was recomputed from the recorded height and weight and agreed with the recorded value in every case (maximum absolute discrepancy 0.005 kg/m^2^). Diabetes duration never equaled or exceeded age. Every dietitian’s total equaled the sum of its component subscores, with or without the age point. The checks and their results are reported in full in Table S9, and the analyses repeated after excluding the records they identified are reported in Table S10. No record was deleted from the primary analysis on the basis of these checks; they are reported transparently and addressed by sensitivity analysis.

### 2.6. Outcomes

The primary outcome was the item-level content of the routine nurse-documented NRS-2002 record, expressed as the proportion of records carrying (i) a score of zero on every item, (ii) a non-zero total attributable solely to the automatically derivable age item, (iii) at least one substantive item (impaired nutritional status or disease severity) scored above zero, and (iv) a total that could not be derived arithmetically from the items recorded alongside it. Category (iv) does not depend on interpretation: a total exceeding the sum of the recorded subscores plus any applicable age point cannot arise from a completed NRS-2002, whatever clinical judgment was exercised. The primary outcome was also summarized as an implementation pathway across all 650 assessed admissions, from form availability to apparently substantive completion.

Three internal validity checks compared the routine record with the patient’s own charted data, independently of the dietitian re-scoring. First, because the initial screen asks whether BMI is below 20.5 kg/m^2^, a charted BMI below this value makes the final screen mandatory, so that in these admissions an all-zero record cannot be explained as a legitimately negative initial screen. Second, because a BMI below 20.5 kg/m^2^ does not by itself compel a non-zero final-screen score (the moderate and severe impaired-nutritional-status categories also require an impaired general condition), a stronger check is restricted to the 35 patients with a BMI below 18.5 kg/m^2^, in whom a non-zero score would ordinarily be expected in a hospitalized patient. Third, we asked whether the presence of any entry varied with charted BMI and with age: because age ≥70 years is not an initial-screen question, a record completed according to the instrument should depend on the initial-screen indications rather than on age.

The secondary outcome was the classification consequence of the routine record: the cross-classification of NRS-2002-defined risk (total ≥3) between the routine record and the standardized re-scoring, summarized principally by the number of re-scored at-risk admissions not identified routinely and by specific agreement. The third, exploratory outcome was the association between non-component clinical and laboratory variables and NRS-2002-defined nutritional risk on standardized re-scoring. Throughout, ‘nutritional risk’ denotes this screening-defined risk and not malnutrition.

### 2.7. Statistical Analysis

All analyses were performed in IBM SPSS Statistics v31, and every statistic reported in this paper is produced by an SPSS procedure, either directly or by arithmetic on its output, with the single exception of the bootstrap optimism correction described below, which was performed in Python 3.12; the procedure behind each statistic is named below. Two-sided *p* < 0.05 was considered statistically significant. Continuous variables are summarized as median (interquartile range [IQR], using the SPSS weighted-average percentile definition) because all key variables departed from normality on the Shapiro–Wilk test (EXAMINE), and categorical variables as *n* (%). Group differences are reported with effect estimates and 95% confidence intervals (CIs) rather than *p* values alone. For continuous variables, the effect estimate is the mean difference with its 95% CI from Welch’s *t*-test (T-TEST, ‘equal variances not assumed’ row), and inference is based on the tie-corrected asymptotic Mann–Whitney *p* value (NPAR TESTS); the median describes the skewed distributions, whereas the mean difference expresses the contrast between groups in clinical units. For proportions, the effect estimate is the risk difference with a Newcombe hybrid-score CI [28], computed from the Wilson limits of the two proportions, and *p* values are from the Pearson χ^2^ test, or Fisher’s exact test when any expected count was below 5 (CROSSTABS). Proportions are reported with Wilson score CIs (One-Sample Proportions), and McNemar’s exact test was obtained from CROSSTABS.

Screening completeness was summarized descriptively with Wilson CIs, overall and within strata (diabetes type, age ≥70 years, ketosis, and length of stay). The implementation pathway across all 650 assessed admissions was summarized with bounds for the 85 admissions whose form content is not available in the analysis dataset. The association of routine documentation (any entry) with age ≥70 years, ketosis, a charted BMI below 20.5 kg/m^2^, sex and a length of stay ≥4 days was examined in an exploratory multivariable logistic model (73 events, five terms); length of stay was included to examine whether early discharge could explain incomplete screening, and diabetes type was omitted because no patient aged ≥70 years had type 1 diabetes. Substantive entries (22 events) were too few for multivariable modeling and were compared descriptively with Fisher’s exact test. Sensitivity of the classification to the re-scoring convention was examined under alternative rules: a literal reading of Kondrup’s table in which every admission receives a disease-severity score of at least 1 for diabetes, with the age point applied unconditionally; the age point applied unconditionally alone; and an extreme lower bound in which the impaired-nutritional-status subscore was disregarded entirely in admissions with a BMI below 20.5 kg/m^2^, so that no point could arise from BMI or from the dietitian’s judgment of general condition.

The cross-classification of risk status is reported as a 2 × 2 table with observed agreement (Wilson CI), positive and negative specific agreement [29] and McNemar’s exact test (CROSSTABS). Cohen’s κ with its asymptotic 95% CI (CROSSTABS) is reported together with the prevalence and bias indices [30,31], to show why it is uninformative here, and was not interpreted against the Landis and Koch descriptive bands [32]: when one source assigns a positive classification to a single patient, κ is constrained almost entirely by the marginal distributions, and describing the result as ‘slight agreement’ would misattribute a documentation failure to rater disagreement [29,31]. Further concordance statistics for the binary, ordinal, and component scores (the prevalence-adjusted bias-adjusted κ, quadratic-weighted κ, intraclass correlation coefficient, exact-score concordance, mean paired difference, Spearman’s ρ, and component-level κ) are reported in Table S13 for completeness; with a single routine positive they add little information and are not interpreted.

Exclusion of scoring components from regression models. BMI and age ≥70 years are constituents of the NRS-2002 total score. Regressing an NRS-2002-derived outcome on these variables is circular: the association is guaranteed by the scoring algorithm, and its magnitude is a property of the instrument, not an empirical finding about patients. Both were therefore excluded on structural grounds from all regression models, and correlations involving them are reported separately and labeled as structural. To quantify what this circularity contributes, a single clearly labeled illustrative model, additionally containing BMI and age, is reported in the Supplementary Materials; it is not offered as a risk model.

The exploratory correlates model was a multivariable logistic regression of NRS-2002-defined risk on sex, diabetes type, diabetes duration, HbA1c, albumin, bicarbonate, and hypertension (124 events for seven terms; 17.7 events per variable [33]). These variables were chosen on clinical grounds as the demographic and diabetes characteristics, the routine admission biochemistry and the most prevalent comorbidity (hypertension, 38.7%); the remaining comorbidities were uncommon (<10%) or, for coronary heart disease, strongly collinear with diabetes type, and infection was defined over a six-month look-back that may include the index illness; infection and coronary heart disease were therefore examined in a sensitivity model. The model was not registered, and we cannot exclude that the choice of variables was influenced by familiarity with the data. Length of stay was excluded because it is determined after admission. Ketosis was excluded from the primary model because decompensated diabetes with ketosis was the dominant qualifying condition for the disease-severity subscore and is therefore partly endogenous to the outcome; it was added in a sensitivity model, and a further analysis was restricted to patients without documented ketosis. HbA1c and bicarbonate are themselves markers of the metabolic decompensation that informs the disease-severity subscore, so their associations with the outcome are partly definitional; excluding ketosis removes the direct variable but not this dependence. The model is therefore interpreted as a description of the clinical profile of re-scored risk, not as evidence of independent associations, and it is not a prediction model: no probability thresholds, sensitivity, specificity or predictive values are reported. Models were fitted in LOGISTIC REGRESSION, with Wald 95% CIs for the odds ratios; unadjusted odds ratios were obtained from univariable models for every candidate variable. Spearman correlations between each study variable and the standardized total score (NONPAR CORR) are reported with 95% CIs from the Fisher z transformation with the Bonett–Wright standard error. Multicollinearity was assessed using variance inflation factors (collinearity diagnostics of the corresponding linear regression), discrimination using the apparent area under the receiver operating characteristic curve (AUC) with its nonparametric 95% CI (ROC), together with a bootstrap estimate of its optimism (1000 resamples of the full model fit), and calibration using the Hosmer–Lemeshow test and a plot of observed against mean predicted risk by decile of predicted probability.

Because the two diabetes types differ substantially in age, adiposity, and metabolic presentation, analyses were stratified by diabetes type, and effect modification was tested with likelihood-ratio tests of diabetes-type interaction terms (the block χ^2^ of LOGISTIC REGRESSION); these six tests were adjusted for multiplicity by the Holm procedure. All regression models were fitted by ordinary maximum likelihood. The type 2 diabetes stratum contains only thirty-five events for six terms (5.8 events per variable), below the conventional threshold [33], so stratum-specific estimates are unstable; they are reported in the Supplementary Materials and are not interpreted individually.

Associations are described throughout as associations or correlates. The terms ‘predictor’, ‘risk factor’, and ‘determinant’ are avoided, since this cross-sectional design cannot support causal or predictive claims. Apart from the six interaction tests, *p* values are unadjusted for multiplicity and are descriptive.

Precision. The sample was fixed by the available records rather than by a formal power calculation. With *N* = 519, the half-width of a 95% Wilson interval for a proportion is at most 4.3 percentage points, and 3.7 percentage points at the observed nutritional-risk prevalence of 23.9%, which was judged adequate for the audit objectives.

## 3. Results

### 3.1. Cohort

Of 650 admission records assessed for eligibility, 131 were excluded—85 because the clinical data required for standardized re-scoring were incomplete and 46 because no baseline NRS-2002 form had been opened at admission—leaving 519 admissions of hospitalized adults with diabetes for analysis (Figure S5). The unit of analysis throughout is the admission, and ‘patients’ below refers to admitted patients counted per admission. The median age was 43 years (IQR 26–62), and 239 patients (46.1%) were male. Type 1 and type 2 diabetes were almost equally represented (258 [49.7%] and 261 [50.3%], respectively), and the median length of stay was 2 days (IQR 1–4). All study variables were complete for all 519 patients with the exception of CRP, available for 111 (21.4%; Table S1).

### 3.2. Completeness and Content of Routine NRS-2002 Documentation

**Figure 1:**
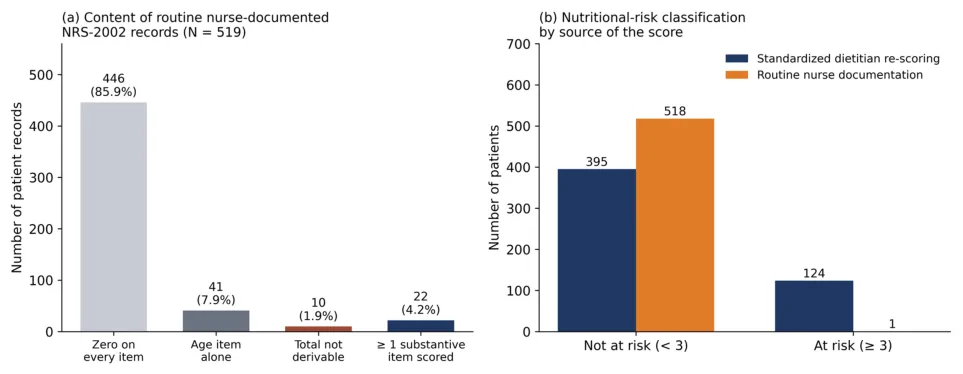
Routine NRS-2002 documentation in 519 hospitalized adults with diabetes. (**a**) Content of the routine nurse-documented record: 446 forms (85.9%) carried a score of zero on every item; 41 (7.9%) carried a non-zero total attributable to the age item alone; 10 (1.9%) carried a non-zero total with both substantive subscores zero in a patient aged under 70, which cannot arise from a completed NRS-2002; and 22 (4.2%) contained at least one substantive item scored above zero. One of the last group also had a total exceeding the sum of its items, giving 11 non-derivable totals in all. (**b**) Nutritional-risk classification by source of the score.

**Figure 2:**
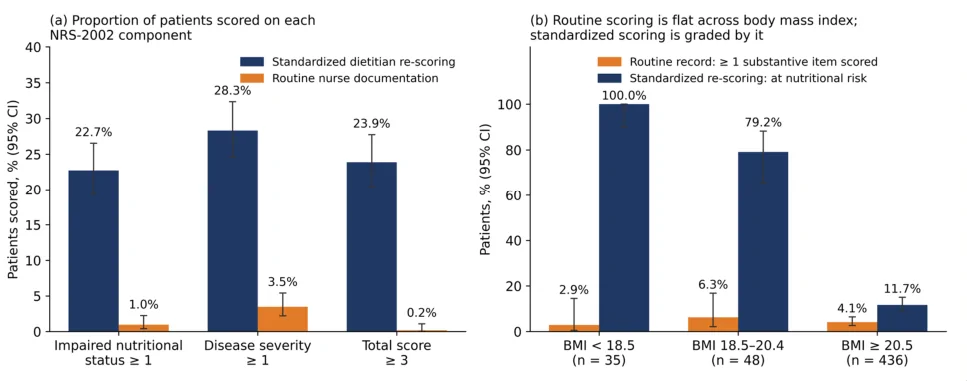
Item-level content and internal consistency of routine NRS-2002 records. (**a**) Proportion of patients scored above zero on each component and classified at risk, by source of the score, with 95% Wilson confidence intervals. (**b**) The same two sources across documented admission body-mass index (BMI). Routine scoring is essentially flat across BMI bands. The gradient in the standardized re-scoring is expected by construction, because BMI is a component of the re-scored subscore, and is shown for reference only; the informative feature is the absence of any gradient in the routine record, which would also be graded by BMI had the instrument been worked through.

Although every included admission had an NRS-2002 form in the record, 446 of 519 forms (85.9%) carried a score of zero on every item. Of the 73 forms with a non-zero total, 41 (56.2%) were attributable solely to the age item, both substantive subscores being zero. Only 22 forms (4.2% of the cohort) contained any substantive item scored above zero: the impaired-nutritional-status subscore exceeded zero in five patients (1.0%, 95% CI 0.4–2.2) and the disease-severity subscore in eighteen (3.5%, 95% CI 2.2–5.4). By comparison, standardized re-scoring assigned a non-zero impaired-nutritional-status subscore to 118 patients (22.7%, 95% CI 19.3–26.5) and a non-zero disease-severity subscore to 147 (28.3%, 95% CI 24.6–32.3). These distributions are shown in Figure 1 and Figure 2a and tabulated in Table 1 (upper panel); the full-score and subscore distributions are given in Table S3.

Across all 650 assessed admissions (Table 1, lower panel), 46 (7.1%) had no NRS-2002 form opened. Of the 565 admissions whose form status was known, 46 (8.1%) had no form, 446 (78.9%) a form carrying zero on every item, 51 (9.0%) a non-zero total without any substantive item (41 from the age item alone and 10 non-derivable), and 22 (3.9%, 95% CI 2.6–5.8) at least one substantive item. The routine form content of the 85 admissions excluded for incomplete clinical data is not available in the analysis dataset; even if every one of them had contained a substantive item, apparently substantive completion would have reached at most 107 of the 650 assessed admissions (16.5%, 95% CI 13.8–19.5).

A subset of the routine totals cannot be reconciled with the items recorded beside them. In 10 records, a non-zero total was accompanied by zero on both substantive subscores in a patient aged under 70, so no age point was available to generate it; in one further record, the total exceeded the sum of the subscores and any applicable age point. These 11 totals (2.1% of forms; 15.1% of the 73 non-zero totals) are too few to carry the central inference on their own. They are reported because they cannot be reconciled with a completed instrument and because they show that the total was entered independently of the subscores rather than computed from them.

The internal validity checks, which rely on the patients’ own charted data rather than on the re-scoring, show that the routine record was insensitive to the indications for screening (Figure 2b). Charted BMI was below 20.5 kg/m^2^ in 83 admissions, in which the initial screen is positive by definition and the final screen mandatory; 79 of these records (95.2%, 95% CI 88.3–98.1) contained no substantive item and 75 (90.4%, 95% CI 82.1–95.0) carried zero on every item. Among the 35 patients with an admission BMI below 18.5 kg/m^2^, a single routine record (2.9%, 95% CI 0.5–14.5) contained any substantive item. Across the whole BMI range the routine record barely moved: a substantive item was scored in 2.9% of patients with a BMI below 18.5, 6.3% of those between 18.5 and 20.4, and 4.1% of those at 20.5 or above. Over the same three bands, standardized re-scoring classified 100%, 79.2% and 11.7% of patients as at nutritional risk, a gradient that is partly built into the re-score because BMI is one of its components. The routine record therefore carried little information about the documented nutritional indications of the patient it describes.

We deliberately restrict the strongest form of this argument to the BMI <18.5 kg/m^2^ group. A BMI below 20.5 kg/m^2^ triggers the final screen but does not compel a non-zero score, since the moderately impaired-nutritional-status category also requires an impaired general condition; consistent with this, the dietitians themselves assigned a zero impaired-nutritional-status subscore to six of the forty-eight patients in the 18.5–20.4 kg/m^2^ band, all of whom had a BMI of 19.1 kg/m^2^ or above. Below 18.5 kg/m^2^, the dietitians exercised no such latitude: every patient in that band received an impaired-nutritional-status subscore of at least 2, which presupposes a judgment of impaired general condition that cannot be verified from the record; the extreme lower-bound rule in Section 3.3 removes this judgment.

Documentation was also selective rather than uniformly absent. Any entry was present in the routine record for 46 of 65 patients aged ≥70 years (70.8%, 95% CI 58.8–80.4) but for only 27 of 454 younger patients (5.9%, 95% CI 4.1–8.5); for 56 of 261 patients with type 2 diabetes (21.5%, 95% CI 16.9–26.8) but 17 of 258 with type 1 diabetes (6.6%, 95% CI 4.2–10.3); and for 13 of 163 patients with ketosis (8.0%, 95% CI 4.7–13.2) versus 60 of 356 without (16.9%, 95% CI 13.3–21.1) (Table S5 and Figure S4). This pattern runs opposite to the indications for the full screen, which were less frequent among older patients: a charted BMI below 20.5 kg/m^2^ was present in four of sixty-five patients aged ≥70 years (6.2%) versus 79 of 454 younger patients (17.4%), and ketosis in none versus 163 (35.9%). In a multivariable model, age ≥70 years was strongly associated with any routine entry (adjusted odds ratio [aOR] 46.6, 95% CI 21.7–100.2), whereas a charted BMI below 20.5 kg/m^2^ was not (aOR 0.85, 95% CI 0.32–2.23), nor were ketosis, sex, or length of stay (Table S15). Substantive entries were similarly rare whether or not re-scoring classified the admission at risk (5.6% vs. 3.8%; *p* = 0.44). Because age ≥70 years is not an initial-screen question, this pattern is more consistent with capture of the one item derivable from the demographic record than with completion triggered by the instrument’s own indications. An individual age-only record may nonetheless be a completed screen with truly zero subscores: in 33 of the 41 such records the re-scoring also assigned zero to both subscores.

Short stays are unlikely to explain the pattern. A substantive item was present in five of one-hundred and forty-seven admissions lasting ≤1 day (3.4%), seven of two-hundred and nine lasting 2–3 days (3.3%) and ten of one-hundred and sixty-three lasting ≥4 days (6.1%), and none of the 36 at-risk admissions lasting ≥4 days was identified routinely (Table S15).

### 3.3. Classification Consequences

Standardized re-scoring classified 124 patients as being at nutritional risk (23.9%, 95% CI 20.4–27.7), whereas the routine record classified one patient (0.2%, 95% CI 0.0–1.1). Of the 124 patients identified by standardized re-scoring, 123 (99.2%) were not identified routinely; disagreement was entirely unidirectional (McNemar exact *p* < 0.001), with no patient classified at risk routinely but not on re-scoring. This result did not depend on the re-scoring convention (Table S14). Awarding every admission a disease-severity point for diabetes, as a literal reading of Kondrup’s table implies, together with an unconditional age point, left the classification of all 519 admissions unchanged (124 at risk). The extreme lower-bound rule, which disregarded the impaired-nutritional-status subscore in the 83 admissions with a BMI below 20.5 kg/m^2^, reduced the number at risk to 57 (11.0%, 95% CI 8.6–14.0), none of whom had been identified routinely.

The cross-classification is given in Table 2. Observed agreement was 0.763 (95% CI 0.725–0.798), but chance-expected agreement was 0.760, so κ was 0.012 (95% CI −0.012 to 0.036); the prevalence index (−0.759) and bias index (−0.237) show that κ here is dominated by the marginal distributions. Negative specific agreement was 0.865, whereas positive specific agreement was 0.016: the two sources concur almost entirely on who is not at risk and essentially not at all on who is. We therefore do not describe this as ‘slight agreement’ between raters; the pattern most plausibly reflects the near-complete absence of a completed screen. Further binary, ordinal, and component concordance statistics are given in Table S13.

At the component level, routine records captured three of the one-hundred and eighteen patients with a non-zero standardized impaired-nutritional-status subscore and six of the one-hundred and forty seven with a non-zero disease-severity subscore; agreement on the ordinal total score was likewise negligible (Table S13).

### 3.4. Characteristics of Patients Classified at Nutritional Risk

Characteristics by NRS-2002-defined risk status on standardized re-scoring are given in Table 3. Patients classified at risk were younger (median 29 vs. 48 years; mean difference −9.85 years, 95% CI −13.97 to −5.74), more often male (54.8% vs. 43.3%; risk difference 11.5 percentage points, 95% CI 1.5 to 21.3) and more often had type 1 diabetes (71.8% vs. 42.8%; risk difference 29.0 points, 95% CI 19.2 to 37.6). They had higher HbA1c (mean difference 1.71%, 95% CI 1.14 to 2.27), lower bicarbonate (−4.81 mmol/L, 95% CI −6.68 to −2.93), a higher prevalence of ketosis (60.5% vs. 22.3%) and a lower prevalence of hypertension (25.0% vs. 43.0%). Serum albumin did not differ between groups (mean difference 0.98 g/L, 95% CI −0.45 to 2.41; *p* = 0.115), nor did CRP in the subset with available data (*p* = 0.128) or length of stay (*p* = 0.248).

BMI and age are reported in Table 3 for descriptive completeness but are marked as scoring components: the difference between groups in these variables is produced in part by the scoring algorithm and is not an independent finding. It is nonetheless relevant that the at-risk group was not simply a group of underweight patients: of the 124 patients classified at risk, 51 (41.1%) had a BMI ≥20.5 kg/m^2^, 30 (24.2%) had a BMI ≥25 kg/m^2^, and 15 (12.1%) had a BMI ≥30 kg/m^2^, so risk was frequently driven by weight loss, reduced intake, and disease severity rather than by low BMI alone.

The contribution of each component to the at-risk classification was as follows. Of the 124 patients classified at risk, 122 (98.4%) had a non-zero disease-severity subscore and 101 (81.5%) a non-zero impaired-nutritional-status subscore; ninety-nine had both, twenty-three reached the threshold through disease severity alone, and two through impaired nutritional status alone. Sixteen at-risk patients were aged ≥70 years, and in eight of them the age point was necessary to reach the threshold of three. The components contributed differently by diabetes type: among at-risk patients with type 1 diabetes, the mean impaired-nutritional-status subscore was 2.19 and the mean disease-severity subscore 1.46, whereas among those with type 2 diabetes, the corresponding means were 1.03 and 1.86 (Table S4). Spearman correlations between each study variable and the standardized NRS-2002 total score, with 95% confidence intervals, are given in Table S8 and Figure S3, in which the scoring components are identified separately because their correlation with the total score is structural.

### 3.5. Differences by Diabetes Type

The two diabetes types differed markedly (Table 4). Patients with type 1 diabetes were younger (median 26 vs. 62 years), leaner (BMI 23.3 vs. 29.6 kg/m^2^), had higher HbA1c (9.7% vs. 8.0%), lower bicarbonate (18.2 vs. 23.7 mmol/L), a far higher prevalence of ketosis (62.0% vs. 1.1%) and a far lower prevalence of hypertension (10.1% vs. 67.0%), and other chronic complications. Nutritional risk on standardized re-scoring was more prevalent in type 1 diabetes (34.5%, 95% CI 29.0–40.5) than in type 2 diabetes (13.4%, 95% CI 9.8–18.1; risk difference 21.1 percentage points, 95% CI 13.8 to 28.1). These differences are the reason all subsequent analyses were stratified by diabetes type, and the reason the younger age of the at-risk group in Table 3 should not be read as an independent finding about age.

### 3.6. Exploratory Clinical and Laboratory Correlates of NRS-2002-Defined Risk

Univariable associations for all candidate variables are given in Table 5. In the exploratory multivariable model, from which the NRS-2002 scoring components were excluded on structural grounds, four variables were associated with NRS-2002-defined risk: higher HbA1c (aOR 1.19 (95% CI 1.08–1.30) per 1%; *p* < 0.001), lower bicarbonate (aOR 0.95 (95% CI 0.92–0.98) per mmol/L; *p* < 0.001), male sex (aOR 1.75 (95% CI 1.11–2.77); *p* = 0.017), and type 1 diabetes (aOR 2.44 (95% CI 1.36–4.37); *p* = 0.003). Diabetes duration showed a borderline inverse association (aOR 0.97 (95% CI 0.95–1.00); *p* = 0.080), and neither serum albumin (aOR 0.99 (95% CI 0.95–1.03); *p* = 0.62) nor hypertension (aOR 1.29 (95% CI 0.68–2.44); *p* = 0.44) was associated. There was no problematic multicollinearity (variance inflation factors 1.06–1.76). The apparent AUC was 0.745 (95% CI 0.695–0.795; optimism-corrected 0.724), Nagelkerke R^2^ was 0.215, and calibration was acceptable (Hosmer–Lemeshow χ^2^ = 6.51, df = 8, *p* = 0.59; Table S7 and Figures S1 and S2). Adding infection and coronary heart disease left the HbA1c (aOR 1.18, 95% CI 1.08–1.30) and bicarbonate (aOR 0.95, 95% CI 0.92–0.98) associations unchanged; coronary heart disease was itself associated with risk (aOR 2.90, 95% CI 1.43–5.88) and the type-1-diabetes association strengthened (aOR 3.45, 95% CI 1.78–6.66), consistent with the collinearity of coronary heart disease with diabetes type (Table S7). Because HbA1c and bicarbonate are themselves markers of the metabolic decompensation that informs the disease-severity subscore, these associations describe the clinical profile of re-scored risk and are not evidence of independent effects.

Quantifying the circularity. Adding BMI and age to the same model raised discrimination from AUC 0.745 (0.695–0.795) to 0.842 (0.796–0.888) and Nagelkerke R^2^ from 0.215 to 0.394, with an apparent BMI effect of 0.81 (95% CI 0.76–0.86) per kg/m^2^. Almost half of the explained variation in a model containing these variables is therefore attributable to constituents of the outcome. This illustrative model (Table S6) is reported to make that contribution explicit and should not be interpreted as evidence that BMI identifies patients at nutritional risk. The adjusted associations for the non-component variables within diabetes-type strata are shown in Figure S6 and Table S11.

### 3.7. Stratified and Sensitivity Analyses

Stratified estimates, interaction tests, and the ketosis sensitivity analyses are reported in Table S11 and Figure S6. HbA1c was associated with NRS-2002-defined risk in both diabetes types (type 1 aOR 1.14, 95% CI 1.02–1.26; type 2 aOR 1.31, 95% CI 1.08–1.58). Two diabetes-type interactions remained significant after Holm adjustment (diabetes duration, Holm-adjusted *p* = 0.021; albumin, Holm-adjusted *p* = 0.029), but because the type-2 stratum contains thirty-five events for six terms, the stratum-specific estimates are unstable and these interactions are regarded as hypothesis-generating only.

Adding ketosis to the primary model attenuated the type 1 diabetes association (aOR 1.49, 95% CI 0.73–3.02), indicating that much of it operates through the acute ketotic state. In the 356 admissions without documented ketosis (49 at risk), HbA1c (aOR 1.23, 95% CI 1.07–1.41) and bicarbonate (aOR 0.92, 95% CI 0.86–0.98) remained associated with NRS-2002-defined risk, and lower albumin was associated with risk only in this subgroup (aOR 0.91, 95% CI 0.86–0.96) (Table S11). Re-scored risk was therefore not confined to ketotic presentations, although HbA1c and bicarbonate may still track metabolic derangement in the absence of documented ketosis.

The data-integrity audit (Section 2.5, Table S9) identified three sets of records warranting sensitivity analysis: seven groups of records identical on all 25 study variables, comprising nine records in excess of one per group, which could not be verified as duplicate entries or genuine repeat admissions because the medical record number was not retained in the de-identified extract; 22 patients with bicarbonate outside 5–45 mmol/L (nineteen below 5 mmol/L, sixteen of them with documented ketosis and compatible with severe ketoacidosis, and three above 45 mmol/L); and five with an implausible implied age at diabetes onset. A fourth analysis excluded the eight at-risk patients who reached the threshold only through the age point. Excluding each set in turn changed no conclusion (Table S10): nutritional-risk prevalence ranged from 22.1% to 24.1% against 23.9% in the primary analysis, and the adjusted odds ratios for HbA1c (1.16–1.19) and bicarbonate (0.95 throughout) remained essentially unchanged and statistically significant. Excluding the identical records left the agreement results identical to three decimal places (κ = 0.012; positive specific agreement 0.016).

## 4. Discussion

In 519 hospital admissions of adults with diabetes, an NRS-2002 form existed in every analyzed record, yet the record rarely showed evidence that the instrument had been worked through. Nearly nine in ten forms carried a score of zero on every item, only one in twenty-four contained even one substantive item scored above zero, and most of the non-zero totals that did exist were generated by the one item that can be filled from the demographic record without seeing the patient; a further 46 admissions had no form at all. When the same admissions were re-scored from the same record using a common written procedure, almost a quarter met the threshold for NRS-2002-defined risk, and 99.2% of these had not been identified in routine practice. The records are therefore strongly suggestive of incomplete implementation of a mandated screen, rather than of disagreement between two groups of clinicians, and this pattern occurred among nurses who had completed mandatory NRS-2002 training and whose access to the screening module depended on holding that competency. Why it occurred cannot be determined from these data.

The evidence for this interpretation is internal to the data and, importantly, does not depend on the dietitian re-scoring. Its strongest element is the insensitivity of the routine record to the indications that the instrument itself uses. A charted BMI below 20.5 kg/m^2^ answers the first initial-screen question affirmatively, so for these 83 admissions the final screen was mandatory and an all-zero form cannot represent a legitimately negative initial screen; yet 79 contained no substantive item. Entries were instead concentrated in patients aged ≥70 years, in whom the indications for the full screen were less, not more, frequent, and age is not an initial-screen question at all. Across ascending BMI bands below 18.5, 18.5–20.4, and 20.5 kg/m^2^ or above, the routine record scored a substantive item in 2.9%, 6.3%, and 4.1% of patients: an instrument that is being worked through should not be flat with respect to a variable it is designed to detect. The eleven arithmetically irreconcilable totals are a smaller, supporting element; too few to carry the inference on their own, they show that totals were entered independently of the items beside them.

Three qualifications apply. First, the extract carries a numeric value for every item, and we could not verify whether the electronic form pre-populates item fields with zero, so a zero deliberately assigned after assessment is indistinguishable from an item never completed, and an all-zero form may represent a legitimately negative initial screen in any admission without a charted indication. If the form does pre-populate zeros, an untouched field and a deliberate zero would be recorded identically; that would be a mechanism of non-completion rather than an alternative to it, and would itself be a target for redesign; nor would it explain why the age item was entered selectively or why totals inconsistent with their subscores were entered. Our inference therefore rests on the pattern across records, not on any single record. Second, an individual age-only record may be a completed screen in an older patient with truly zero subscores—the re-scoring itself assigned zero to both subscores in 33 of the 41—although the concentration of such records in older patients, who less often met the indications for the full screen, is not what protocol-driven completion would produce. Third, a zero impaired-nutritional-status subscore in the 18.5–20.4 kg/m^2^ band is a legitimate score, since the moderate category also requires an impaired general condition; our own dietitians assigned zero to six of the forty-eight patients in that band. Conversely, under the literal reading of Kondrup’s table in which diabetes itself carries a disease-severity score of one [12], every mandatory final screen in this cohort should carry a disease-severity score of at least one, yet 82 of the 83 forms for which charted BMI made the final screen mandatory (98.8%, 95% CI 93.5–99.8) carried zero.

This framing has a direct consequence for how the agreement statistics should be read. A κ of 0.012 in these data is not a measure of how much two raters disagreed; it is an arithmetical consequence of one source assigning a positive classification to a single patient out of 519. The observed agreement of 0.763 and the prevalence-adjusted bias-adjusted κ of 0.526 (Table S13) describe the same table and appear far more favorable, while positive specific agreement—the quantity that actually matters when the purpose of screening is case-finding—was 0.016. These are the classical κ paradoxes arising under extreme prevalence and marginal imbalance [29,31], and reporting κ alone against descriptive bands such as ‘slight agreement’ [32] would obscure rather than convey what happened. We would suggest that studies of screening implementation report screening completion rates and specific agreement as primary quantities, with κ, if reported at all, accompanied by the prevalence and bias indices.

The findings sit at the extreme end of a consistent literature. Paulsen et al. reported low inter-rater reliability between nurses and researchers using the NRS-2002 (κ = 0.27) [20], and Deschepper et al. only 72% agreement between nurses and dietitians (κ = 0.44), stressing the danger of falsely labeling patients as not at risk [21]; trained and calibrated assessors, by contrast, achieve κ values of 0.65–0.81 [34] and test–retest agreement as high as 0.956 [35]. Geurden et al. and Geiker et al. documented that the parameters required for screening are frequently absent from nursing records [22,23], and Thomsen et al. showed that routine screening in a university hospital was insufficient and underestimated nutritional risk [24]. The distinguishing feature here is the magnitude: not attenuated agreement but its near-total absence, most plausibly reflecting non-completion.

Two recent studies frame that result sharply, because both concern nurses who had been trained in the instrument. In the VAL-NUT study, Canta et al. reported that trained nurses screened 248 patients admitted to internal medicine and orthopedics and identified 36 (14.5%) at nutritional risk, concluding that training and empowerment enable nurses to identify and manage patients at risk effectively [25]. Zhang et al., studying 434 hospitalized patients with cancer screened by 35 trained nurses against a dietitian reference, reported fair overall agreement (κ = 0.343) but a striking gradient across the instrument’s items: perfect for the age item (κ = 1.000), fair for impaired nutritional status (κ = 0.241), and poor for disease severity (κ = 0.198) [26]. Our data show the same gradient in a more extreme form: the item requiring no patient contact was by far the most often captured. The gradient may therefore reflect not only the conceptual difficulty of the disease-severity construct, as Zhang et al. suggest, but also the amount of bedside work each item demands.

Nurses in this setting were also trained: NRS-2002 competency is a mandatory institutional requirement and access to the electronic screening module is restricted to nurses who hold it. This makes a complete absence of training an unlikely explanation, but it does not establish that knowledge was adequate, because we could not assess the quality, recency or retention of training, practical competency, staffing, workload, competing clinical priorities, supervision or the design of the electronic interface. The contrast with VAL-NUT is nonetheless instructive: those nurses were trained and were also working inside a study, with a protocol, a defined screening window and a post-training evaluation [25], whereas ours were working inside ordinary practice. Workflow and accountability structures, such as mandatory fields, automated prompts, feedback, and audit, are therefore plausible contributors alongside knowledge retention and competency, consistent with evidence that structured protocols and training together improve screening performance [36,37], that embedding the tool in the electronic record improves case identification and referral [38,39,40], that nutrition support teams improve consistency of care [41], and that competing priorities and workload influence whether screening happens [42,43]. On this evidence, training may be necessary but, as delivered, did not ensure completion; which of these factors operated here can only be established prospectively.

A second contribution of this analysis is methodological. Previous work has modeled NRS-2002-defined nutritional risk as a function of BMI. BMI is a constituent of the impaired-nutritional-status subscore, and age ≥70 years contributes a point to the total; regressing the score on them is therefore circular, and the resulting coefficients describe the scoring algorithm rather than the patients. The magnitude of this artifact is not trivial: in the present data, adding BMI and age to the model raised the AUC from 0.745 to 0.842 and nearly doubled the Nagelkerke R^2^, and the apparent BMI ‘effect’ of 0.81 per kg/m^2^ is essentially a restatement of the NRS-2002 BMI thresholds. Reported associations between BMI and screening-tool-defined nutritional risk [44,45,46] should be read with this in mind. The same caution applies, less absolutely, to variables that inform the disease-severity component: in this cohort decompensated diabetes with ketosis was the dominant qualifying condition, so ketosis is partly endogenous to the outcome, which is why we excluded it from the primary model and examined it separately.

Once the scoring components were removed, the associations that remained were metabolic rather than anthropometric: higher HbA1c and lower bicarbonate, with male sex and type 1 diabetes. Because HbA1c and bicarbonate also mark the metabolic decompensation that informs the disease-severity subscore, these associations are partly definitional and describe the clinical profile of re-scored risk rather than independent findings. Higher HbA1c has been reported in association with nutritional risk in hospitalized patients with diabetes by Chang et al. [16], whereas Thaenpramun et al. found no association between glycemic control and Mini Nutritional Assessment scores in ambulatory older adults with type 2 diabetes [47]—a discrepancy plausibly explained by differences in acuity, since HbA1c in an acutely decompensated inpatient marks a period of catabolic, insulin-deficient metabolism rather than diet quality alone [48]. Lower bicarbonate is likewise consistent with Chang et al. [16] and with the known relationship between ketone concentrations, acidosis, and severity of decompensation [49], and concurrent malnutrition in diabetic ketoacidosis is associated with worse inpatient outcomes [50]. The reasonable reading is that in this population an NRS-2002 score of ≥3 often marks acute metabolic decompensation with attendant catabolic stress, which is a legitimate indication for nutritional attention, rather than established chronic undernutrition.

The apparent association between nutritional risk and younger age deserves explicit comment, because it is counterintuitive. It is almost entirely an artifact of case mix. Patients with type 1 diabetes were 36 years younger at the median, were much more often ketotic, and had more than twice the prevalence of nutritional risk. Age is also a scoring component in the opposite direction, which makes its unadjusted association with the total score uninterpretable. In the component-excluded models, diabetes type rather than age carried the association, and adding ketosis attenuated it further. We therefore do not interpret younger age as a marker of nutritional risk in hospitalized diabetes.

Some associations may differ between diabetes types: diabetes duration and albumin showed interactions that remained significant after Holm adjustment, but the type 2 stratum provides fewer than six events per variable, so we treat these as hypothesis-generating. Albumin did not differ between risk groups in the whole cohort (*p* = 0.115) but was lower in at-risk patients without ketosis, consistent with the established behavior of albumin as an acute-phase reactant governed by inflammation, capillary leak, hydration, and hepatic synthesis rather than by protein-energy stores [51,52,53], with hemoconcentration plausibly masking the relationship in ketotic patients. Albumin is unlikely to be a reliable nutritional marker in acutely decompensated patients.

Several questions cannot be answered from these data. We had no reference-standard diagnosis of malnutrition—neither the GLIM criteria nor Subjective Global Assessment was recorded—so we cannot state what proportion of patients were malnourished, nor evaluate the criterion validity of the NRS-2002 here. The 23.9% reported is a record-based estimate of NRS-2002-defined risk, sensitive to the scoring convention (11.0–23.9% across the rules in Table S14), and not a prevalence of malnutrition; in a Saudi cohort with type 2 diabetes GLIM showed substantial agreement with Subjective Global Assessment [54], and a comparable validation is the necessary next step. We also had no outcome data, so we cannot show that the patients missed by routine screening fared worse, although the association between nutritional risk and mortality, length of stay and readmission is well established [5,6,55,56,57] and nutritional support in patients at risk improves outcomes [58,59,60,61]. Finally, the laboratory panel was limited to albumin, HbA1c, bicarbonate and CRP; a fuller biochemical characterization would be informative in future work.

### 4.1. Strengths and Limitations

The principal strengths are the size of the cohort for a single-center screening audit; the availability of item-level rather than only total scores for both sources, which permitted the component and internal-consistency analyses that underpin our interpretation; the availability of charted indications, which allowed the principal checks to be made independently of the re-scoring; the fact that all routine scores were entered by nurses who had completed mandatory institutional NRS-2002 training and held credentialed access to the screening module, which makes a complete absence of training an unlikely explanation for the findings; and the exclusion of scoring components from all regression models on structural grounds.

The limitations are substantial. First and most importantly, the extract carries a numeric value for every NRS-2002 item and does not include the initial-screen responses, item-entry timestamps or the audit trail of the electronic form, and we could not verify whether item fields default to zero. It therefore cannot distinguish an item never completed from one deliberately scored zero, nor a legitimately negative initial screen from one never performed; non-completion is an inference from the pattern of the data—mandatory final screens without substantive entries, the concentration of entries on the age item, the flatness of the record with respect to BMI and the arithmetically irreconcilable totals—not a direct observation. A prospective audit with timestamped item-level entry and direct observation would settle it. Second, the analytic identifier denotes admissions and the medical record number was not retained in the de-identified extract, so repeat admissions of the same patient could not be identified or modeled; seven groups of records were identical across all 25 study variables (nine records in excess of one per group) and could not be verified against source records, although excluding them changed nothing (Table S10). Clustering by ward, nurse, shift or calendar period, including the COVID-19 pandemic and any changes to the form or the electronic record, could not be examined because these variables were not extracted; the descriptive proportions are unaffected, but their CIs may be somewhat narrow if admissions are clustered. Third, the design is not an inter-rater reliability study: the two sets of scores were produced under different conditions by assessors who were neither blinded nor independent, and no statement about the competence of nurses relative to dietitians is warranted. Fourth, the dietitian re-scoring was retrospective and record-based: it measures documented information rather than patient status, the number of patients with documented weight-change and intake data was not captured (although at least 47 admissions received impaired-nutritional-status points that could only have arisen from such documentation), and the judgments of impaired general condition and of disease severity in ketotic presentations had no objective threshold and cannot be audited. Each admission was re-scored by a single dietitian whose identity was not recorded, and none was double-scored, so the reliability of the re-scoring could not be estimated and it should not be regarded as a validated reference standard. The procedure was not registered or time-stamped, so it cannot be shown to have been fixed before re-scoring began. The dietitians were also not blinded to the routine entry: knowledge of a recorded value may have anchored the re-score toward it, which would tend to inflate apparent agreement, while the predominance of all-zero routine records may have cued a benign impression and so understated risk. The 23.9% therefore depends on the scoring convention (Table S14), although the near-complete non-recognition of re-scored risk did not, and the principal evidence of non-completion is read from the routine record and charted data alone. Fifth, the cohort was restricted to admissions with an NRS-2002 form and sufficient clinical data. The 46 admissions excluded because no form had been opened are the most unequivocal expression of non-implementation, and the 85 excluded for incomplete clinical data may include further admissions in which screening was not performed; across all 650 assessed admissions, apparently substantive completion lies between 3.4% and 16.5% (Table 1), and estimates confined to the analytic cohort are likely to be optimistic. Selection on data availability also explains the unusually complete variables in the analytic dataset. Sixth, the study is retrospective, single-center, and conducted in a population with an unusually high proportion of type 1 diabetes and ketotic presentations; many admissions were short (median 2 days), and the route of admission was not extracted, although substantive entries remained rare in admissions lasting 4 days or longer. Seventh, there is no reference-standard nutritional diagnosis and no outcome data. Eighth, CRP was available for 21.4% of patients and its analyses are exploratory. Ninth, the regression analyses are exploratory: HbA1c and bicarbonate are partly definitional correlates of the outcome, the type-2 stratum contains only 35 events, and only the six interaction tests were adjusted for multiplicity. Tenth, although training and credentialed access are mandatory, we could not measure the recency or retention of each nurse’s training, competency, nurse identity, staffing, acuity or workload, so the causes of the observed pattern cannot be determined. Eleventh, no protocol was registered and the audit framing was adopted after initial inspection of the records, so all analyses should be regarded as descriptive. Finally, the cross-sectional design supports associations only.

### 4.2. Implications

For practice, the findings indicate a substantial implementation and documentation problem but do not establish its cause: workflow and interface design, workload, knowledge retention, organizational culture, and other factors may each have contributed. The pattern nonetheless suggests specific quality-improvement strategies that could be tested prospectively: making the substantive items mandatory before the admission record can be completed, auto-populating BMI from charted height and weight so that a BMI below 20.5 kg/m^2^ prompts the full screen, generating an automatic dietitian referral when the threshold is met, giving ward teams feedback, and auditing completion as a routine quality indicator, ideally alongside refresher training and competency assessment so that their relative contributions can be evaluated. For research, we would encourage reporting of screening completion alongside agreement statistics, reporting of the implementation pathway from form availability to substantive completion, and the exclusion of scoring components from any model of screening-tool-defined risk.

## 5. Conclusions

In this retrospective audit of 519 hospital admissions of adults with diabetes, routine NRS-2002 documentation was strongly suggestive of incomplete implementation, although the forms were entered by nurses with mandatory training in the instrument and credentialed access to it: 85.9% of routine forms carried a score of zero on every item, only 4.2% contained any substantive item, 11 of the 73 non-zero totals could not be derived from the items beside them, 95.2% of the forms for which charted BMI made the full screen mandatory contained no substantive item, and a further 46 admissions had no form at all. Standardized record-based re-scoring classified 23.9% of admissions at NRS-2002-defined risk, of whom 99.2% had not been identified routinely, and this near-complete non-recognition persisted under alternative scoring rules. The records cannot show why screening was incomplete. Workflow-embedded completion, automated referral, feedback, and routine audit are reasonable strategies to be evaluated prospectively, alongside attention to knowledge retention and competency. Models of screening-defined risk should exclude the instrument’s own components. These findings do not establish the prevalence of malnutrition, which requires validation against a reference standard such as the GLIM criteria, nor the clinical consequences of the missed cases, which requires linkage to outcomes.

## Figures and Tables

**Table 1:** Item-level content of routine nurse-documented NRS-2002 records, with standardized dietitian re-scoring of the same admissions for comparison (N = 519; upper panel), and implementation pathway across all 650 assessed admissions (lower panel).

Item	Routine Record, *n* (%)	95% CI (%)	Standardized Re-Scoring, *n* (%)	95% CI (%)
Score of zero on every NRS-2002 item	446 (85.9)	82.7–88.7	368 (70.9)	66.9–74.6
Non-zero total from the age item alone	41 (7.9)	5.9–10.5	1 (0.2)	0.0–1.1
Total not derivable from the recorded items †	11 (2.1)	1.2–3.8	0 (0.0)	0.0–0.7
≥1 substantive item scored above zero	22 (4.2)	2.8–6.3	150 (28.9)	25.2–32.9
Impaired nutritional status ≥1	5 (1.0)	0.4–2.2	118 (22.7)	19.3–26.5
Disease severity ≥1	18 (3.5)	2.2–5.4	147 (28.3)	24.6–32.3
Total score ≥3 (classified at nutritional risk)	1 (0.2)	0.0–1.1	124 (23.9)	20.4–27.7
Implementation pathway across all 650 assessed admissions				
No NRS-2002 form opened	46 (7.1)	5.3–9.3	—	—
Form content not available (excluded: incomplete clinical data) ‡	85 (13.1)	10.7–15.9	—	—
Form with a score of zero on every item	446 (68.6)	64.9–72.1	—	—
Non-zero total without any substantive item	51 (7.8)	6.0–10.2	—	—
≥1 substantive item scored above zero	22 (3.4)	2.2–5.1	—	—
Upper bound for substantive completion ‡	107 (16.5)	13.8–19.5	—	—

CI, confidence interval (Wilson). The age item contributes one point for age ≥70 years and is derivable from the demographic record without patient contact; it is therefore not counted as a substantive item. Categories are not mutually exclusive below the first two rows. In one standardized record the age point was recorded with both substantive subscores at zero (Section 2.4). † A total exceeding the sum of the recorded subscores plus any applicable age point, which cannot arise from a completed NRS-2002. The extract carries a numeric value for every item, so a score deliberately assigned as zero cannot be distinguished from an item never completed (Section 2.4). Standardized re-scoring was performed by dietitians from the same admission record using a common written procedure; it is record-based and not a validated reference standard. ‡ Routine form content is not available in the analysis dataset for the 85 admissions excluded because clinical data required for re-scoring were incomplete; the bound assumes that every one of them contained a substantive item.

**Table 2:** Cross-classification of NRS-2002-defined nutritional risk (total ≥ 3) between routine documentation and standardized dietitian re-scoring (N = 519).

Statistic	Value (95% CI)
Cross-classification, n	
At risk on both	1
At risk routinely only	0
At risk on standardized re-scoring only	123
Not at risk on either	395
Binary classification (NRS-2002 ≥ 3)	
Observed agreement	0.763 (0.725–0.798)
Chance-expected agreement	0.760
Cohen’s κ (asymptotic CI)	0.012 (−0.012 to 0.036)
Prevalence index	−0.759
Bias index	−0.237
Positive specific agreement	0.016
Negative specific agreement	0.865
McNemar exact test	*p* < 0.001

CI, confidence interval. The prevalence index quantifies the imbalance between the two diagonal cells and the bias index the imbalance between the two off-diagonal cells; both indicate that κ here is dominated by the marginal distributions. These statistics describe the classification consequence of a routine record against a record-based re-score of the same admission; they are not estimates of inter-rater reliability (Section 2.1). Specific agreement is shown as a point estimate. Further concordance statistics, including the prevalence-adjusted bias-adjusted κ, and the ordinal and component-level statistics, are given in Table S13.

**Table 3:** Characteristics of the cohort by nutritional-risk status defined by standardized NRS-2002 re-scoring (N = 519).

Characteristic	Overall (*N* = 519)	Not At Risk (*n* = 395)	At Risk (*n* = 124)	Difference (95% CI)	*p*
Demographics					
Age (years) †	43.0 (26.0–62.0)	48.0 (30.0–63.0)	29.0 (22.0–53.8)	−9.85 (−13.97 to −5.74)	<0.001
Male sex, n (%)	239 (46.1)	171 (43.3)	68 (54.8)	11.5 (1.5 to 21.3)	0.024
Clinical					
Type 1 diabetes, n (%)	258 (49.7)	169 (42.8)	89 (71.8)	29.0 (19.2 to 37.6)	<0.001
Duration of diabetes (years)	13.0 (8.0–20.0)	14.0 (9.0–20.0)	10.0 (4.3–17.0)	−3.43 (−5.25 to −1.62)	<0.001
Length of stay (days)	2.0 (1.0–4.0)	2.0 (1.0–5.0)	2.0 (1.0–4.0)	−0.55 (−1.44 to 0.33)	0.248
Anthropometry					
BMI (kg/m^2^) †	27.1 (22.1–32.0)	28.5 (24.2–33.1)	19.6 (18.2–24.8)	−7.25 (−8.56 to −5.94)	<0.001
Laboratory					
Albumin (g/L)	40.3 (35.6–44.0)	40.3 (35.6–43.7)	40.6 (35.2–46.1)	0.98 (−0.45 to 2.41)	0.115
HbA1c (%)	8.5 (7.2–10.6)	8.2 (7.0–10.0)	10.0 (8.1–12.4)	1.71 (1.14 to 2.27)	<0.001
Bicarbonate (mmol/L)	22.1 (16.5–25.5)	23.0 (18.4–26.0)	17.3 (9.1–22.9)	−4.81 (−6.68 to −2.93)	<0.001
CRP (mg/L) ‡	16.4 (3.5–121.8)	23.5 (5.3–142.3)	14.6 (2.9–39.0)	−40.88 (−89.49 to 7.72)	0.128
Comorbidities and complications, n (%)					
Ketosis	163 (31.4)	88 (22.3)	75 (60.5)	38.2 (28.4 to 47.2)	<0.001
Infection (prior 6 months)	112 (21.6)	79 (20.0)	33 (26.6)	6.6 (−1.6 to 15.8)	0.118
Coronary heart disease	97 (18.7)	73 (18.5)	24 (19.4)	0.9 (−6.4 to 9.5)	0.828
Hypertension	201 (38.7)	170 (43.0)	31 (25.0)	−18.0 (−26.4 to −8.5)	<0.001
Diabetic kidney disease	25 (4.8)	21 (5.3)	4 (3.2)	−2.1 (−5.4 to 3.0)	0.343
Neuropathy	16 (3.1)	11 (2.8)	5 (4.0)	1.2 (−1.9 to 6.5)	0.551
Retinopathy	46 (8.9)	38 (9.6)	8 (6.5)	−3.2 (−7.7 to 3.1)	0.279

Continuous variables are median (IQR) per group, with the mean difference (at risk minus not at risk) and its 95% CI from Welch’s *t*-test; *p* values are the asymptotic two-tailed Mann–Whitney U *p* values, on which inference is based. Medians and IQRs use the SPSS weighted-average percentile definition; the median describes these skewed distributions, and the mean difference expresses the contrast in clinical units. Categorical variables are n (%) with the risk difference in percentage points and its Newcombe 95% CI; *p* values from the χ^2^ or Fisher’s exact test as appropriate. † NRS-2002 scoring component: the between-group difference is generated in part by the scoring algorithm and is shown for description only. ‡ CRP available for 111 patients (21.4%). BMI, body mass index; CI, confidence interval; CRP, C-reactive protein; IQR, interquartile range.

**Table 4:** Characteristics by diabetes type (N = 519).

Variable	Type 1 Diabetes (*n* = 258)	Type 2 Diabetes (*n* = 261)	Difference (95% CI)	*p*
Age (years)	26.0 (22.0–33.0)	62.0 (52.0–69.5)	−33.42 (−35.29 to −31.56)	<0.001
Duration of diabetes (years)	11.0 (5.0–17.0)	15.0 (10.0–20.0)	−3.79 (−5.32 to −2.27)	<0.001
BMI (kg/m^2^)	23.3 (20.0–28.9)	29.6 (26.2–34.1)	−6.12 (−7.23 to −5.01)	<0.001
HbA1c (%)	9.7 (7.5–11.6)	8.0 (7.0–9.6)	1.36 (0.94 to 1.77)	<0.001
Albumin (g/L)	41.2 (36.0–45.1)	39.7 (35.3–43.2)	1.31 (0.25 to 2.36)	0.009
Bicarbonate (mmol/L)	18.2 (11.0–24.0)	23.7 (20.7–26.2)	−4.40 (−5.72 to −3.08)	<0.001
CRP (mg/L)	13.8 (3.5–24.3)	25.0 (4.0–153.4)	−70.39 (−114.56 to −26.22)	0.035
Length of stay (days)	2.0 (1.0–3.0)	3.0 (2.0–6.0)	−2.84 (−3.64 to −2.03)	<0.001
Male sex	109 (42.2)	130 (49.8)	−7.6 (−16.0 to 1.0)	0.084
Ketosis	160 (62.0)	3 (1.1)	60.9 (54.4 to 66.6)	<0.001
Hypertension	26 (10.1)	175 (67.0)	−57.0 (−63.2 to −49.7)	<0.001
Coronary heart disease	7 (2.7)	90 (34.5)	−31.8 (−37.9 to −25.6)	<0.001
Diabetic kidney disease	6 (2.3)	19 (7.3)	−5.0 (−9.0 to −1.3)	0.008
Retinopathy	11 (4.3)	35 (13.4)	−9.1 (−14.2 to −4.3)	<0.001
Neuropathy	2 (0.8)	14 (5.4)	−4.6 (−8.1 to −1.7)	0.002
Infection (prior 6 months)	52 (20.2)	60 (23.0)	−2.8 (−9.9 to 4.3)	0.433
Nutritional risk (NRS-2002 ≥ 3)	89 (34.5)	35 (13.4)	21.1 (13.8 to 28.1)	<0.001

Continuous variables are median (IQR) per group, with the mean difference (type 1 minus type 2) and its 95% CI from Welch’s *t*-test and the asymptotic Mann–Whitney U *p* value; categorical variables are n (%) with the risk difference in percentage points and its Newcombe 95% CI. CRP available for 111 patients. CI, confidence interval; CRP, C-reactive protein; IQR, interquartile range.

**Table 5:** Exploratory univariable and multivariable associations with NRS-2002-defined nutritional risk on standardized re-scoring (N = 519; 124 events).

Variable	Unadjusted OR (95% CI)	*p*	Adjusted OR (95% CI)	*p*
Male sex	1.59 (1.06–2.39)	0.025	1.75 (1.11–2.77)	0.017
Type 1 diabetes	3.40 (2.19–5.27)	<0.001	2.44 (1.36–4.37)	0.003
Duration of diabetes (per year)	0.95 (0.93–0.98)	<0.001	0.97 (0.95–1.00)	0.080
HbA1c (per 1%)	1.30 (1.20–1.42)	<0.001	1.19 (1.08–1.30)	<0.001
Albumin (per 1 g/L)	1.03 (0.99–1.06)	0.122	0.99 (0.95–1.03)	0.619
Bicarbonate (per 1 mmol/L)	0.92 (0.90–0.95)	<0.001	0.95 (0.92–0.98)	<0.001
Hypertension	0.44 (0.28–0.69)	<0.001	1.29 (0.68–2.44)	0.436
Ketosis	5.34 (3.47–8.22)	<0.001	—	—
Infection (prior 6 months)	1.45 (0.91–2.32)	0.120	—	—
Coronary heart disease	1.06 (0.63–1.77)	0.828	—	—
Diabetic kidney disease	0.59 (0.20–1.76)	0.348	—	—
Neuropathy	1.47 (0.50–4.31)	0.486	—	—
Retinopathy	0.65 (0.29–1.43)	0.282	—	—
Length of stay (per day)	0.97 (0.93–1.02)	0.271	—	—
CRP (per 1 mg/L)	1.00 (0.99–1.00)	0.132	—	—
NRS-2002 scoring components (not eligible for the model) †				
Age (per year)	0.97 (0.96–0.98)	<0.001	Excluded (structural)	—
BMI (per 1 kg/m^2^)	0.79 (0.75–0.83)	<0.001	Excluded (structural)	—

Adjusted estimates are from a single multivariable-logistic model containing sex, diabetes type, diabetes duration, HbA1c, albumin, bicarbonate, and hypertension (apparent AUC 0.745, 95% CI 0.695–0.795; optimism-corrected 0.724). Length of stay was not eligible because it is determined after admission; ketosis was excluded from the primary model because it is partly endogenous to the disease-severity subscore (see Table S11 for the sensitivity model including it); CRP was not eligible because it was available for 111 patients only. † BMI and age ≥70 years are constituents of the NRS-2002 total score; unadjusted estimates are shown to document the structural association and are not interpretable as risk associations. *p* values are unadjusted for multiplicity. CI, confidence interval; OR, odds ratio.

## Data Availability

The de-identified admission-level dataset is available from the corresponding author on reasonable request.

## Supplementary Materials

The following supporting information can be downloaded at: https://www.mdpi.com/article/10.3390/healthcare14203527/s1, Supplementary Methods S1–S3 (reconstruction of the NRS-2002 from the two sources; agreement and concordance statistics; regression modeling); Table S1, completeness of study variables; Table S2, verification of the NRS-2002 age-adjustment convention; Table S3, distribution of NRS-2002 total scores and component subscores by source; Table S4, contribution of each component to the at-risk classification, overall and by diabetes type; Table S5, screening completion and detected nutritional risk across clinical strata; Table S6, illustrative model containing the NRS-2002 scoring components, reported to quantify circularity; Table S7, discrimination, internal validation, and calibration of the exploratory correlates model, with a sensitivity model; Table S8, Spearman correlations with 95% confidence intervals, with scoring components identified separately; Table S9, data-quality and integrity audit; Table S10, sensitivity of the principal results to the records identified by that audit; Table S11, stratified analyses by diabetes type, interaction tests, and ketosis-sensitivity analyses; Table S12, distribution of standardized disease-severity subscores across documented conditions; Table S13, additional concordance statistics; Table S14, sensitivity of the risk classification to alternative scoring rules; Table S15, routine documentation by clinical indication, length of stay, and re-scored risk, with a multivariable model of any entry; Figure S1, receiver-operating characteristic curves; Figure S2, calibration plot; Figure S3, correlation plot; Figure S4, screening completion and detected risk by stratum; Figure S5, participant flow; Figure S6, adjusted associations within diabetes-type strata.
