# Supplementary material for: Documented but Not Demonstrably Performed: A Retrospective Audit of Routine NRS-2002 Screening Records in Hospitalized Adults with Diabetes
Source: Healthcare (Basel). 2026 Oct 20;14(20):3527. doi: 10.3390/healthcare14203527 (PMC13704705; doi:10.3390/healthcare14203527)
Supplement: Supplementary file 1 [file healthcare-14-03527-s001.zip › healthcare-4618819-supplementary.pdf]

Supplementary Material

Documented but Not Demonstrably Performed: A Retrospective Audit of Routine NRS-2002 Screening Records in Hospitalized Adults with Diabetes

This file contains Supplementary Methods S1–S3, Supplementary Tables S1–S15 and Supplementary Figures S1–S6. All values were computed in IBM SPSS Statistics v31 from the de-identified analysis dataset (N = 519 admissions), except the bootstrap optimism correction in Table S7, which was computed in Python 3.12.

Supplementary Methods

S1. Reconstruction of the NRS-2002 from the two sources

Routine scores were extracted verbatim from the admission screening form, retaining the impaired-nutritional-status subscore, the disease-severity subscore and the recorded total as three separate fields. No routine value was altered, imputed or corrected at any stage. Records carrying a score of zero on every item were retained as recorded. All routine scores were entered by nursing staff who had completed the institution's mandatory NRS-2002 training; access to the electronic screening module is restricted to nurses holding that competency, so every record analyzed was produced by a trained, credentialed user of the instrument. The quality, recency and retention of that training were not measured. The extract did not include the initial-screen responses, item timestamps or the audit trail of the electronic form, and whether item fields default to zero could not be verified; because 11 recorded totals exceed the sum of their subscores and any applicable age point, the total is not computed automatically from the subscores.

Standardized re-scoring was performed by clinical dietitians from the same electronic record using a common written procedure; "standardized" denotes this common procedure, not a validated reference standard, and because the patients were not examined the re-score reflects documented information. The impaired-nutritional-status subscore was assigned from admission BMI together with documented weight change and documented oral intake in the preceding week, following the published NRS-2002 categories; impaired general condition, which the moderate and severe BMI categories require, was judged from the documented clinical state at admission. The disease-severity subscore was assigned from the documented admission diagnosis and acute clinical condition using the operational definitions of Kondrup et al. (score 1, chronic disease with complications and a modest increase in requirements; score 2, a patient confined to bed by illness; score 3, illness of intensive-care severity); no point was awarded for the diagnosis of diabetes alone, and ketotic presentations were graded by clinical severity without a laboratory threshold (Table S12). Each admission was re-scored by one of three dietitians (173 each, allocated at random), whose identity was not recorded in the analysis dataset; no admission was double-scored, and the dietitians were not blinded to the routine entry. Laboratory values (albumin, HbA1c, bicarbonate, CRP) are not NRS-2002 components; no laboratory cut-off was used to assign or modify any item, and these variables were analyzed only as descriptive correlates.

Age adjustment. The NRS-2002 adds one point for age ≥ 70 years. In the study dataset the age point was applied to 17 of the 65 patients aged ≥ 70 years: to all 16 with at least one substantive subscore above zero, and to one patient in whom both substantive subscores were zero (total score 1, not at risk). In the remaining 48 elderly patients both substantive subscores were zero and no age point was added, consistent with the NRS-2002 structure in which the final screen is reached only after a positive initial screen. Recomputing every total as (impaired nutritional status + disease severity + age point), applying the age point unconditionally, yields 124 patients at risk versus 124 as recorded, and changes the risk classification of 0 patients (Table S2). The convention therefore has no bearing on any result.

Alternative scoring rules. To test whether the classification depends on the re-scoring convention, totals were recomputed under three alternative rules (Table S14): (A) a literal reading of Kondrup's disease-severity table, under which every admission of a patient with diabetes receives a disease-severity score of at least 1, with the age point applied unconditionally; (B) the age point applied unconditionally with the subscores as recorded (Table S2); and (C) an extreme lower bound in which the impaired-nutritional-status subscore was set to zero in all 83 admissions with a BMI below 20.5 kg/m², so that no point could arise from BMI or from the judgment of impaired general condition (weight-loss and intake points in these admissions were also removed, which makes rule C deliberately conservative). Rule C combined with rule A was also examined. A dietitian-independent check counted the admissions in which a charted BMI below 20.5 kg/m² made the final screen mandatory.

S2. Agreement and concordance statistics

Because the marginal distributions were extremely asymmetric, no single agreement index is adequate. Observed agreement is the proportion of patients classified identically by the two sources, reported with its Wilson score CI. Positive and negative specific agreement are 2a/(2a+b+c) and 2d/(2d+b+c) respectively, where a and d are the concordant positive and negative cells and b and c the discordant cells; they answer the question 'given that one source classified a patient positive (negative), how often did the other agree?'. Cohen's κ is reported with the Fleiss–Cohen–Everitt asymptotic standard error, which is the estimator SPSS CROSSTABS implements. The prevalence index is (a − d)/n and the bias index is (b − c)/n; the prevalence-adjusted bias-adjusted κ (PABAK) equals $2p_0 - 1$, so its CI is obtained by applying the same transformation to the Wilson limits of p_0. Specific agreement is reported as a point estimate. Ordinal concordance was summarized by the quadratic-weighted κ with its asymptotic CI (WEIGHTED KAPPA procedure), the two-way random-effects absolute-agreement single-measurement intraclass correlation coefficient (RELIABILITY), exact score concordance and the mean paired difference with its t-based interval. Directional disagreement was tested with the exact binomial form of McNemar's test. Only the cross-classification, observed agreement, specific agreement, McNemar's test and κ with its prevalence and bias indices are reported in the main text; the remaining statistics are given in Table S13.

S3. Regression modeling

BMI and age ≥ 70 years are constituents of the NRS-2002 total score and were excluded on structural grounds from all regression models; their unadjusted associations are reported only to document the structural relationship. Length of stay was excluded because it is determined after admission. Ketosis was excluded from the primary model because decompensated diabetes with ketosis was the

dominant qualifying condition for the disease-severity subscore and is therefore partly endogenous to the outcome; it was examined in a sensitivity model and in a ketosis-free subgroup. CRP was not eligible for multivariable modeling (available for 111 patients) and was evaluated descriptively. The exploratory correlates model contained sex, diabetes type, diabetes duration, HbA1c, albumin, bicarbonate and hypertension (124 events, 17.7 events per variable). These variables were chosen on clinical grounds as the demographic and diabetes characteristics, the routine admission biochemistry and the most prevalent comorbidity; the other comorbidities were uncommon (< 10%) or, for coronary heart disease, strongly collinear with diabetes type, and infection was defined over a six-month look-back that may include the index illness. The model was not registered. HbA1c and bicarbonate are markers of the metabolic decompensation that informs the disease-severity subscore, so their associations are partly definitional, and the model is not a prediction model. Its apparent AUC was corrected for optimism by Harrell's bootstrap method (1000 resamples of the full model fit; seed 2026), and a sensitivity model added infection and coronary heart disease (Table S7). Correlates of routine documentation were examined with a logistic model of any routine entry on age ≥ 70 years, ketosis, BMI < 20.5 kg/m², sex and length of stay ≥ 4 days (73 events; Table S15). Effect modification by diabetes type was tested by adding an interaction term to the primary model and comparing nested models by likelihood-ratio test (block χ^2 in LOGISTIC REGRESSION); the six tests were adjusted by the Holm procedure and are interpreted as hypothesis-generating. The type 2 diabetes stratum contains 35 events for six terms (5.8 events per variable), so its estimates are unstable (Table S11). Other P values are unadjusted and descriptive. Missing data were not imputed.

Supplementary Tables

Table S1. Completeness of study variables (N = 519).

Variable	Available, n	Missing, n (%)
Age	519	0 (0.0)
Duration of diabetes	519	0 (0.0)
Length of stay	519	0 (0.0)
BMI	519	0 (0.0)
HbA1c	519	0 (0.0)
Albumin	519	0 (0.0)
Bicarbonate	519	0 (0.0)
C-reactive protein	111	408 (78.6)
Sex	519	0 (0.0)
Diabetes type	519	0 (0.0)
Ketosis	519	0 (0.0)
Infection (prior 6 months)	519	0 (0.0)
Coronary heart disease	519	0 (0.0)
Hypertension	519	0 (0.0)
Diabetic kidney disease	519	0 (0.0)
Neuropathy	519	0 (0.0)
Retinopathy	519	0 (0.0)
NRS-2002 items (both sources)	519	0 (0.0)

Only C-reactive protein had missing values. All demographic, clinical, anthropometric and remaining biochemical variables, and all NRS-2002 items from both sources, were complete for all 519 patients.

Table S2. Verification of the NRS-2002 age-adjustment convention.

Check	Result
Patients aged ≥ 70 years	65
Age point applied in the dataset	17
Of which ≥ 1 substantive subscore > 0 / both subscores zero	16 / 1
Elderly patients with both subscores zero and no age point	48
Patients at risk, as recorded	124
Patients at risk, recomputing the total with the age point applied unconditionally	124
Patients whose risk classification changes under the alternative convention	0

Table S3. Distribution of NRS-2002 total scores and component subscores, by source.

Score	Total score, routine	Total score, standardized	Impaired status, routine	Impaired status, standardized	Disease severity, routine	Disease severity, standardized
0	446 (85.9)	368 (70.9)	514 (99.0)	401 (77.3)	501 (96.5)	372 (71.7)
1	63 (12.1)	9 (1.7)	3 (0.6)	29 (5.6)	16 (3.1)	107 (20.6)
2	9 (1.7)	18 (3.5)	2 (0.4)	48 (9.2)	2 (0.4)	5 (1.0)
3	1 (0.2)	66 (12.7)	0 (0.0)	41 (7.9)	0 (0.0)	35 (6.7)
4	0 (0.0)	49 (9.4)	—	—	—	—
5	0 (0.0)	6 (1.2)	—	—	—	—
6	0 (0.0)	3 (0.6)	—	—	—	—

Total scores range 0–7 and subscores 0–3. A total score ≥ 3 denotes nutritional risk.

Table S4. Contribution of each NRS-2002 component to the at-risk classification (standardized re-scoring; n = 124 at risk).

Contribution	All at risk (n = 124)	Type 1 diabetes (n = 89)	Type 2 diabetes (n = 35)
Impaired nutritional status ≥ 1, n (%)	101 (81.5)	79 (88.8)	22 (62.9)
Disease severity ≥ 1, n (%)	122 (98.4)	87 (97.8)	35 (100.0)
Both components ≥ 1, n	99	—	—
Impaired nutritional status only, n	2	—	—
Disease severity only, n	23	—	—
Mean impaired-nutritional-status subscore	1.86	2.19	1.03
Mean disease-severity subscore	1.57	1.46	1.86
Aged ≥ 70 years (age point applied), n	16	0	16

Contribution	All at risk (n = 124)	Type 1 diabetes (n = 89)	Type 2 diabetes (n = 35)
Reached the threshold of 3 only because of the age point, n	8	—	—

Body mass index distribution within the at-risk group: < 18.5 kg/m², n = 35; 18.5–20.4 kg/m², n = 38; ≥ 20.5 kg/m², n = 51 (41.1%), of whom 30 had a body mass index ≥ 25 kg/m² and 15 ≥ 30 kg/m². Nutritional risk was therefore frequently driven by weight loss, reduced intake and disease severity rather than by low body mass index alone.

Table S5. Screening completion and detected nutritional risk across clinical strata.

Stratum	n	Any entry in the routine record, n (%) [95% CI]	At risk on standardized re-scoring, n (%)	At risk identified routinely, n	Not identified routinely, n (%)
Type 1 diabetes	258	17 (6.6) [4.2–10.3]	89 (34.5)	0	89 (100.0)
Type 2 diabetes	261	56 (21.5) [16.9–26.8]	35 (13.4)	1	34 (97.1)
Age < 70 years	454	27 (5.9) [4.1–8.5]	108 (23.8)	0	108 (100.0)
Age ≥ 70 years	65	46 (70.8) [58.8–80.4]	16 (24.6)	1	15 (93.8)
Ketosis present	163	13 (8.0) [4.7–13.2]	75 (46.0)	0	75 (100.0)
No ketosis	356	60 (16.9) [13.3–21.1]	49 (13.8)	1	48 (98.0)

CI, confidence interval (Wilson).

Table S6. Illustrative model containing the NRS-2002 scoring components — reported only to quantify circularity, and not interpretable as a risk model.

Variable	Adjusted OR (95% CI)	P
Male sex	1.23 (0.73–2.04)	0.437
Type 1 diabetes	1.83 (0.64–5.27)	0.260
Duration of diabetes (per year)	0.97 (0.93–1.00)	0.051
HbA1c (per 1%)	1.15 (1.04–1.27)	0.007
Albumin (per 1 g/L)	0.97 (0.94–1.01)	0.192
Bicarbonate (per 1 mmol/L)	0.95 (0.93–0.98)	0.003
Hypertension	1.37 (0.66–2.86)	0.400
BMI (per 1 kg/m2) †	0.81 (0.76–0.86)	<0.001
Age (per year) †	1.02 (0.99–1.05)	0.171
Model AUC (95% CI)	0.842 (0.796–0.888)	
Nagelkerke R^2	0.394	
Primary (component-excluded) model, AUC (95% CI)	0.745 (0.695–0.795)	
Primary (component-excluded) model, Nagelkerke R^2	0.215	

† NRS-2002 scoring component. Adding body mass index and age to the primary model raises discrimination from AUC 0.745 to 0.842 and Nagelkerke R^2 from 0.215 to 0.394. This gain is generated by the scoring algorithm rather than by any independent association, which is why these variables were excluded from all reported models.

Table S7. Discrimination, internal validation and calibration of the exploratory (component-excluded) correlates model, and a sensitivity model adding infection and coronary heart disease.

Statistic	Exploratory model (7 terms)	Sensitivity model (+ infection, CHD)
Apparent AUC (95% CI)	0.745 (0.695–0.795)	0.768 (0.721–0.815)
Optimism-corrected AUC (bootstrap)	0.724	0.743
Optimism-corrected calibration slope	0.91	—
Nagelkerke R^2	0.215	0.243
Hosmer–Lemeshow χ^2 (df), P	6.51 (8), 0.59	—
Variance inflation factors	1.06–1.76	—
Male sex, aOR (95% CI)	1.75 (1.11–2.77)	1.60 (1.00–2.55)
Type 1 diabetes, aOR (95% CI)	2.44 (1.36–4.37)	3.45 (1.78–6.66)
Duration of diabetes, per year	0.97 (0.95–1.00)	0.97 (0.95–1.00)
HbA1c, per 1%	1.19 (1.08–1.30)	1.18 (1.08–1.30)
Albumin, per 1 g/L	0.99 (0.95–1.03)	1.00 (0.96–1.04)
Bicarbonate, per 1 mmol/L	0.95 (0.92–0.98)	0.95 (0.92–0.98)
Hypertension	1.29 (0.68–2.44)	1.09 (0.56–2.12)
Infection (prior 6 months)	—	1.58 (0.94–2.65)
Coronary heart disease	—	2.90 (1.43–5.88)

aOR, adjusted odds ratio; AUC, area under the receiver operating characteristic curve; CHD, coronary heart disease; CI, confidence interval. The model describes the clinical profile of re-scored risk and is not a prediction model; operating characteristics at probability cut-offs (sensitivity, specificity and predictive values) are therefore not reported. Apparent AUC with nonparametric 95% CI (SPSS ROC). Optimism was estimated by refitting the model in 1000 bootstrap resamples and averaging the difference between the AUC in the resample and in the original data (Harrell FE, Lee KL, Mark DB. Stat. Med. 1996, 15, 361–387); the same procedure gave the calibration slope. Calibration plot in Figure S2. For comparison, the illustrative model additionally containing body mass index and age (Table S6) showed significant miscalibration (Hosmer–Lemeshow $P < 0.001$).

Table S8. Spearman correlations between study variables and the standardized NRS-2002 total score, with 95% confidence intervals.

Variable	n	ρ (95% CI)	P
Duration of diabetes (years)	519	−0.140 (−0.224 to −0.054)	0.001
Length of stay (days)	519	−0.088 (−0.173 to −0.001)	0.046
HbA1c (%)	519	0.239 (0.155 to 0.319)	<0.001
Albumin (g/L)	519	0.116 (0.030 to 0.201)	0.008
Bicarbonate (mmol/L)	519	−0.270 (−0.349 to −0.187)	<0.001
CRP (mg/L)	111	−0.153 (−0.331 to 0.035)	0.108
Male sex	519	0.098 (0.012 to 0.183)	0.025
Type 1 diabetes	519	0.308 (0.226 to 0.385)	<0.001
Ketosis	519	0.416 (0.339 to 0.488)	<0.001
Infection (prior 6 months)	519	0.076 (−0.010 to 0.161)	0.083
Coronary heart disease	519	−0.029 (−0.115 to 0.057)	0.513

Variable	n	ϱ (95% CI)	P
Hypertension	519	−0.200 (−0.282 to −0.115)	<0.001
Diabetic kidney disease	519	−0.049 (−0.134 to 0.037)	0.266
Neuropathy	519	0.039 (−0.047 to 0.125)	0.376
Retinopathy	519	−0.060 (−0.145 to 0.026)	0.173
NRS-2002 scoring components †			
Age (years) †	519	−0.270 (−0.349 to −0.186)	<0.001
BMI (kg/m²) †	519	−0.542 (−0.604 to −0.473)	<0.001

Confidence intervals from the Fisher z transformation with the Bonett–Wright standard error, as provided by the SPSS Bivariate Correlations procedure (NONPAR CORR). Bicarbonate and age have the same ϱ to three decimals (−0.2698 and −0.2696); their intervals differ in the third decimal. † These variables are constituents of the NRS-2002 total score; their correlation with it is structural and is shown for completeness only.

Table S9. Data-quality and integrity audit of the analysis dataset (N = 519).

Check	Result	Action taken
Duplicate admission identifier (Patient_ID)	0	—
Records identical on all 25 study variables	7 groups (sizes 3, 3, 2, 2, 2, 2, 2); 9 records in excess of one per group	Retained; sensitivity analysis (Table S10); source verification not possible because the medical record number was not retained in the de-identified extract
BMI recomputed from height and weight	Agreed in 519/519 (max |difference| 0.005 kg/m²)	—
Diabetes duration ≥ age	0	—
Implied age at onset < 1 year	0	—
Type 2 diabetes with implied onset age < 10 years	1	Retained; sensitivity analysis
Type 1 diabetes with implied onset age > 40 years	4	Retained; sensitivity analysis
Bicarbonate outside 5–45 mmol/L	22 (range 2.0–50.0): 19 below 5 mmol/L (16 with documented ketosis), 3 above 45 mmol/L	Retained; sensitivity analysis
Age > 100 years	1 (105 years)	Retained
Length of stay < 1 day	1	Retained
Height, weight, BMI, HbA1c, albumin, CRP out of plausible range	0	—
Dietitian total inconsistent with its own subscores	0	—
Dietitian subscore outside 0–3	0	—
Routine total below the sum of its subscores	0	—
Routine total above subscore sum + age point (impossible)	11 (15.1% of the 73 non-zero totals)	Reported as supporting evidence of non-completion (Section 3.2)
Routine subscore outside 0–3	0	—
BMI < 18.5 with dietitian impaired-status subscore < 2	0	—

Check	Result	Action taken
BMI 18.5–20.4 with dietitian impaired-status subscore = 0	6 of 48	Reported in Section 3.2; strong claim restricted to BMI < 18.5

The extract carries a numeric value for every NRS-2002 item, so a score deliberately assigned as zero cannot be distinguished from an item that was never completed; this is stated as the first limitation of the study. No record was removed from the primary analysis on the basis of these checks. Bicarbonate values below 5 mmol/L are physiologically compatible with severe diabetic ketoacidosis, which is defined by a bicarbonate concentration below 10 mmol/L (Kitabchi AE, Umpierrez GE, Miles JM, Fisher JN. Diabetes Care 2009, 32, 1335–1343); values above 45 mmol/L are extreme and were retained but examined in Table S10. The analytic identifier denotes admissions; whether identical records are duplicate entries or repeat admissions of the same patient could not be determined.

Table S10. Sensitivity of the principal results to the records identified by the data-quality audit.

Result	Primary (N = 519)	Identical records removed	Age-point-only cases excluded	Extreme bicarbonate excluded	Implausible onset excluded
n / events	519 / 124	510 / 123	511 / 116	497 / 110	514 / 123
Male sex, aOR (95% CI)	1.75 (1.11–2.77)	1.79 (1.12–2.84)	1.84 (1.13–2.98)	1.77 (1.11–2.82)	1.77 (1.11–2.80)
Type 1 diabetes, aOR (95% CI)	2.44 (1.36–4.37)	2.50 (1.39–4.51)	2.99 (1.62–5.55)	2.23 (1.23–4.03)	2.50 (1.39–4.52)
Duration of diabetes, aOR (95% CI)	0.97 (0.95–1.00)	0.98 (0.95–1.00)	0.97 (0.94–1.00)	0.98 (0.95–1.01)	0.97 (0.95–1.00)
HbA1c, aOR (95% CI)	1.19 (1.08–1.30)	1.19 (1.09–1.31)	1.19 (1.09–1.31)	1.16 (1.06–1.28)	1.19 (1.08–1.30)
Albumin, aOR (95% CI)	0.99 (0.95–1.03)	1.00 (0.96–1.03)	0.99 (0.95–1.03)	0.99 (0.95–1.03)	0.99 (0.95–1.03)
Bicarbonate, aOR (95% CI)	0.95 (0.92–0.98)	0.95 (0.92–0.97)	0.95 (0.92–0.98)	0.95 (0.92–0.98)	0.95 (0.92–0.98)
Hypertension, aOR (95% CI)	1.29 (0.68–2.44)	1.32 (0.70–2.52)	1.15 (0.59–2.28)	1.28 (0.67–2.43)	1.34 (0.70–2.56)
Model AUC	0.745	0.751	0.774	0.722	0.746
Nutritional-risk prevalence, % (95% CI)	23.9 (20.4–27.7)	24.1 (20.6–28.0)	22.7 (19.3–26.5)	22.1 (18.7–26.0)	23.9 (20.4–27.8)

aOR, adjusted odds ratio; AUC, area under the receiver operating characteristic curve; CI, confidence interval. Each column repeats the primary multivariable model after excluding the records named in the column heading. No conclusion changes. Removing the nine records in excess of one per identical group also left the agreement results unchanged (κ = 0.012, positive specific agreement 0.016, all-zero routine records 86.1%, 122 of 123 at-risk patients unrecognized).

Table S11. Stratified analyses by diabetes type, diabetes-type interaction tests, and ketosis sensitivity analyses.

Variable	Type 1 diabetes (n = 258; 89 events)	Type 2 diabetes (n = 261; 35 events)	Interaction P (Holm)	Without ketosis (n = 356; 49 events)	Whole cohort + ketosis (n = 519; 124 events)

Variable	Type 1 diabetes (n = 258; 89 events)	Type 2 diabetes (n = 261; 35 events)	Interaction P (Holm)	Without ketosis (n = 356; 49 events)	Whole cohort + ketosis (n = 519; 124 events)
Male sex	1.45 (0.80–2.64)	1.91 (0.86–4.25)	0.986 (0.986)	1.26 (0.65–2.45)	1.74 (1.10–2.77)
Duration of diabetes (per year)	0.94 (0.91–0.98)	1.01 (0.97–1.06)	0.004 (0.021)	1.00 (0.96–1.03)	0.98 (0.95–1.01)
HbA1c (per 1%)	1.14 (1.02–1.26)	1.31 (1.08–1.58)	0.257 (0.514)	1.23 (1.07–1.41)	1.14 (1.04–1.26)
Albumin (per 1 g/L)	1.03 (0.98–1.08)	0.95 (0.89–1.01)	0.006 (0.029)	0.91 (0.86–0.96)	0.97 (0.93–1.01)
Bicarbonate (per 1 mmol/L)	0.97 (0.94–1.00)	0.89 (0.83–0.95)	0.014 (0.056)	0.92 (0.86–0.98)	0.96 (0.94–0.99)
Hypertension	0.84 (0.27–2.56)	1.72 (0.66–4.45)	0.049 (0.147)	1.28 (0.60–2.74)	1.48 (0.75–2.90)
Type 1 diabetes	—	—	—	1.12 (0.51–2.48)	1.49 (0.73–3.02)
Ketosis	—	—	—	—	2.98 (1.43–6.20)
Model AUC (95% CI)	0.721 (0.655–0.788)	0.783 (0.707–0.858)	—	0.738 (0.664–0.812)	0.767 (0.720–0.815)

All values are adjusted odds ratios (95% CI) from multivariable logistic models excluding the NRS-2002 scoring components, fitted by ordinary maximum likelihood. The type 2 diabetes stratum contains 35 events, so its estimates are imprecise and are interpreted with caution. Interaction P values are from likelihood-ratio tests of a diabetes-type interaction term added to the primary model, with Holm-adjusted values in parentheses for the six tests performed; they are exploratory. AUC, area under the receiver operating characteristic curve; CI, confidence interval. With 35 events for six terms (5.8 events per variable), the type 2 diabetes estimates are unstable and are not interpreted individually; the interaction tests are hypothesis-generating.

Table S12. Distribution of standardized disease-severity subscores across documented conditions (N = 519).

Documented condition	n	Score 0	Score 1	Score 2	Score 3
**All admissions**	519	372	107	5	35
Type 1 diabetes	258	150	84	3	21
Type 2 diabetes	261	222	23	2	14
**Ketosis documented**	163	73	72	2	16
Ketosis: bicarbonate < 10 mmol/L	52	14	28	0	10
Ketosis: bicarbonate 10–14.9 mmol/L	43	25	17	0	1
Ketosis: bicarbonate 15–17.9 mmol/L	13	8	4	0	1
Ketosis: bicarbonate ≥ 18 mmol/L	55	26	23	2	4
**No ketosis documented**	356	299	35	3	19
No ketosis: infection in prior 6 months	72	50	16	0	6
No ketosis: no infection	284	249	19	3	13

Scores were assigned from the documented admission diagnosis and acute clinical condition using Kondrup's operational definitions; no laboratory threshold was used, and the bicarbonate bands are shown for description only. No point was awarded for the diagnosis of diabetes alone. The dataset does not contain the free-text diagnoses on which the scores were based, so the distribution illustrates, rather than reconstructs, the dietitians' decisions; it also shows the judgment involved (for example, 14 ketotic admissions with bicarbonate < 10 mmol/L

were scored 0). Table S14 shows that the risk classification is unchanged when every admission is awarded a disease-severity point for diabetes.

Table S13. Additional concordance statistics between routine documentation and standardized re-scoring (N = 519).

Statistic	Value (95% CI)
**Binary classification (NRS-2002 ≥ 3)**	
Prevalence-adjusted bias-adjusted κ (PABAK)	0.526 (0.449–0.595)
**Ordinal total score (0–7)**	
Weighted κ, quadratic (asymptotic CI)	0.024 (−0.017 to 0.066)
Intraclass correlation coefficient (2,1)	0.024 (−0.046 to 0.096)
Spearman's ϱ, P	0.022, P = 0.62
Exact score concordance	0.618
Mean difference, routine − standardized	−0.78 (−0.91 to −0.64)
**Component subscores (any vs none)**	
Impaired nutritional status, κ	0.031 (−0.013 to 0.075)
Disease severity, κ	0.012 (−0.038 to 0.062)

PABAK equals $2p_0 - 1$, so its CI follows from the Wilson CI of observed agreement. With a single admission classified at risk routinely, these statistics add little information beyond the cross-classification and specific agreement and are not interpreted; they are not estimates of inter-rater reliability.

Table S14. Sensitivity of the NRS-2002-defined risk classification to alternative scoring rules (N = 519).

Scoring rule	At risk, n (%) [95% CI]	Identified routinely, n	Not identified routinely, n (%) [95% CI]
Primary (standardized re-scoring as recorded)	124 (23.9) [20.4–27.7]	1	123 (99.2) [95.6–99.9]
A. Literal disease-severity rule (diabetes ≥ 1 for every admission) and unconditional age point	124 (23.9) [20.4–27.7]	1	123 (99.2) [95.6–99.9]
B. Unconditional age point only (Table S2)	124 (23.9) [20.4–27.7]	1	123 (99.2) [95.6–99.9]
C. Extreme lower bound: impaired-nutritional-status subscore set to 0 when BMI < 20.5 kg/m²	57 (11.0) [8.6–14.0]	0	57 (100.0) [93.7–100.0]
C combined with A	57 (11.0) [8.6–14.0]	0	57 (100.0) [93.7–100.0]
**Dietitian-independent check**			
Charted BMI < 20.5 kg/m² (final screen mandatory)	83 (16.0) [13.1–19.4] †	4 with a substantive item	79 (95.2) [88.3–98.1] without a substantive item

Rule A changes no classification because every admission not already at risk that had a disease-severity subscore of 0 had an impaired-nutritional-status subscore plus any age point below 2, so the added point brings none of them to the threshold of 3. Rule C removes all points that could arise from BMI, from the judgment of impaired general condition, and also from weight loss or reduced intake in the affected admissions; it is therefore a

deliberately extreme lower bound. † Number of admissions with a mandatory final screen, not a number at risk. Wilson 95% CIs. The routine record classified one admission at risk under every rule.

Table S15. Routine NRS-2002 documentation by clinical indication for the final screen, length of stay and re-scored risk, with a multivariable model of any routine entry (N = 519).

Characteristic	n	Any entry, n (%) [95% CI]	Substantive item, n (%) [95% CI]	Any entry, aOR (95% CI)
Age ≥ 70 years	65	46 (70.8) [58.8–80.4]	5 (7.7) [3.3–16.8]	46.6 (21.7–100.2)
Age < 70 years	454	27 (5.9) [4.1–8.5]	17 (3.7) [2.4–5.9]	Reference
BMI < 20.5 kg/m² (final screen mandatory)	83	8 (9.6) [5.0–17.9]	4 (4.8) [1.9–11.7]	0.85 (0.32–2.23)
BMI ≥ 20.5 kg/m²	436	65 (14.9) [11.9–18.6]	18 (4.1) [2.6–6.4]	Reference
Ketosis documented	163	13 (8.0) [4.7–13.2]	9 (5.5) [2.9–10.2]	2.18 (0.90–5.31)
No ketosis	356	60 (16.9) [13.3–21.1]	13 (3.7) [2.1–6.1]	Reference
Male	239	41 (17.2) [12.9–22.4]	13 (5.4) [3.2–9.1]	1.49 (0.79–2.82)
Female	280	32 (11.4) [8.2–15.7]	9 (3.2) [1.7–6.0]	Reference
Length of stay ≤ 1 day	147	19 (12.9) [8.4–19.3]	5 (3.4) [1.5–7.7]	—
Length of stay 2–3 days	209	19 (9.1) [5.9–13.8]	7 (3.3) [1.6–6.8]	—
Length of stay ≥ 4 days	163	35 (21.5) [15.9–28.4]	10 (6.1) [3.4–10.9]	1.72 (0.85–3.45) ‡
At risk on standardized re-scoring	124	19 (15.3) [10.0–22.7]	7 (5.6) [2.8–11.2]	—
Not at risk on standardized re-scoring	395	54 (13.7) [10.6–17.4]	15 (3.8) [2.3–6.2]	—

Any entry denotes a non-zero routine total; a substantive item denotes a non-zero impaired-nutritional-status or disease-severity subscore. The multivariable model (73 events; five terms) contained age ≥ 70 years, BMI < 20.5 kg/m², ketosis, sex and length of stay ≥ 4 days (‡ versus < 4 days); diabetes type was omitted because no patient aged ≥ 70 years had type 1 diabetes. Substantive entries (22 events) were too few for modeling; Fisher's exact P for substantive entry: age 0.18, BMI < 20.5 kg/m² 0.77, ketosis 0.35, sex 0.28, length of stay ≥ 4 days 0.16, re-scored risk 0.44. None of the 36 at-risk admissions lasting ≥ 4 days was identified routinely. Indications for the final screen were less frequent in patients aged ≥ 70 years (BMI < 20.5 kg/m²: 6.2% vs 17.4%; ketosis: 0% vs 35.9%). aOR, adjusted odds ratio; CI, confidence interval (Wilson).

Supplementary Figures

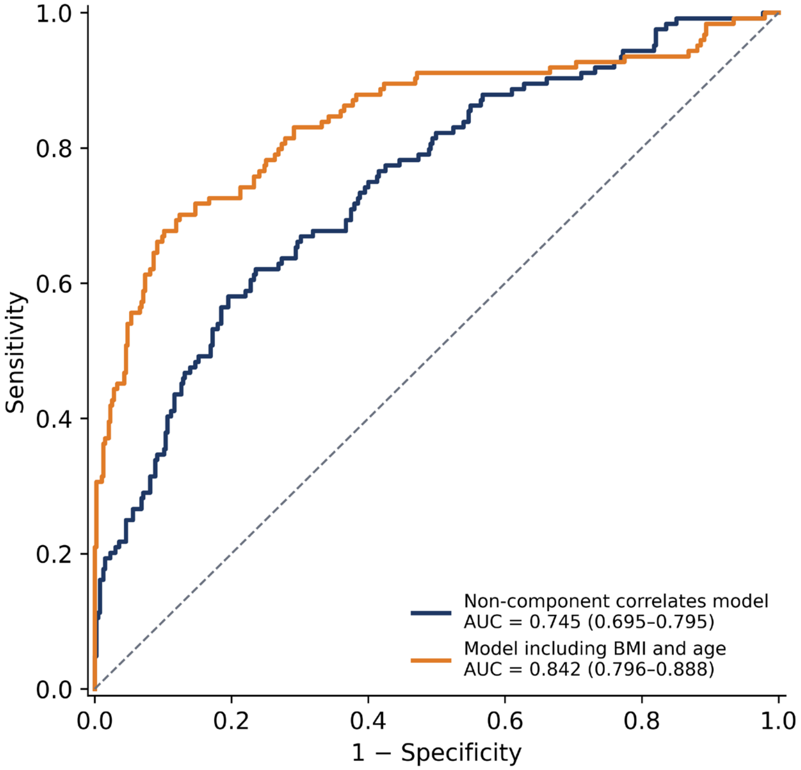

Figure S1. Receiver operating characteristic curves for the exploratory component-excluded model (apparent AUC 0.745, 95% CI 0.695–0.795; optimism-corrected 0.724) and for the same model with body mass index and age added (AUC 0.842, 95% CI 0.796–0.888); nonparametric 95% CIs. The difference quantifies the discrimination attributable to variables that are constituents of the outcome. Neither curve represents a validated prediction model.

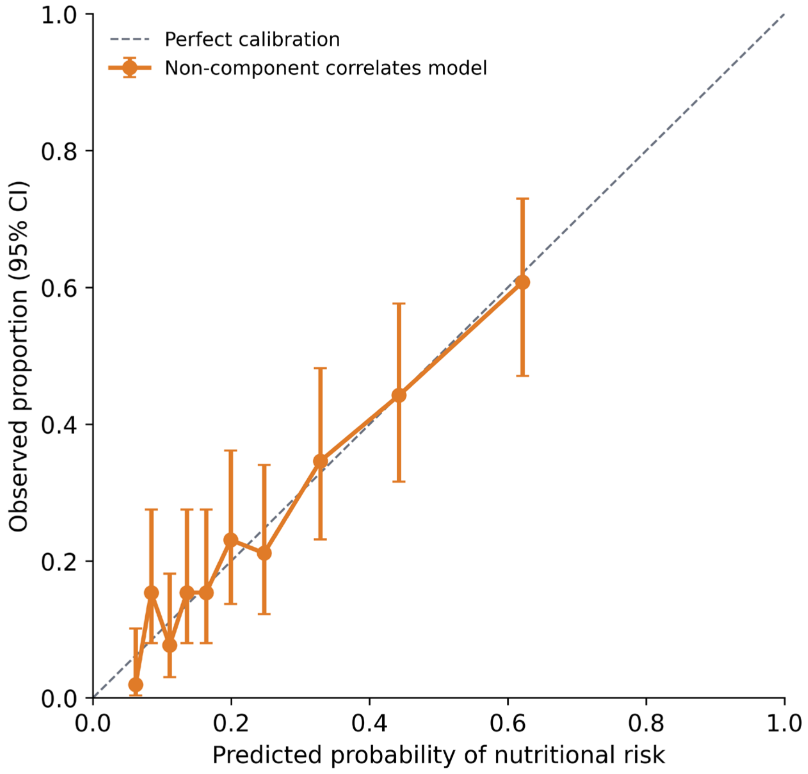

Figure S2. Calibration plot for the exploratory component-excluded model, by decile of predicted probability, with 95% Wilson confidence intervals (Hosmer–Lemeshow P = 0.59).

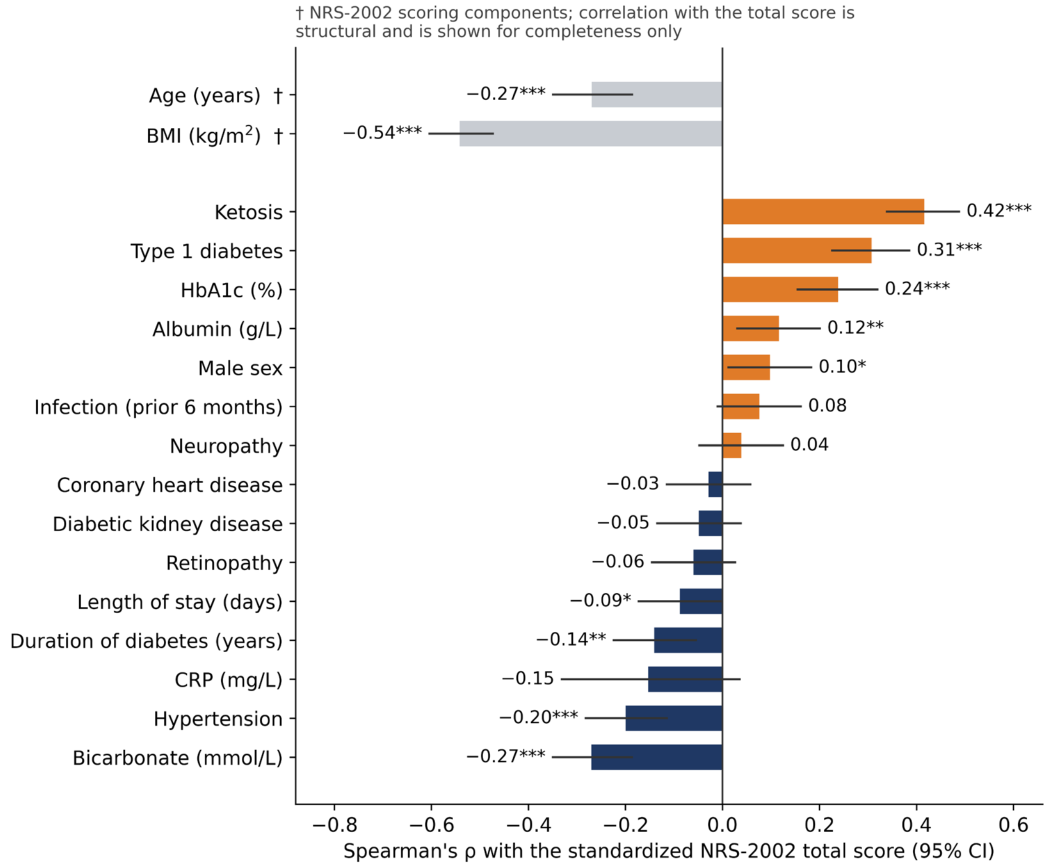

Figure S3. Spearman correlations between study variables and the standardized NRS-2002 total score, with 95% confidence intervals. NRS-2002 scoring components are shown separately in gray and marked †; their correlation with the total score is structural. *P < 0.05; **P < 0.01; ***P < 0.001.

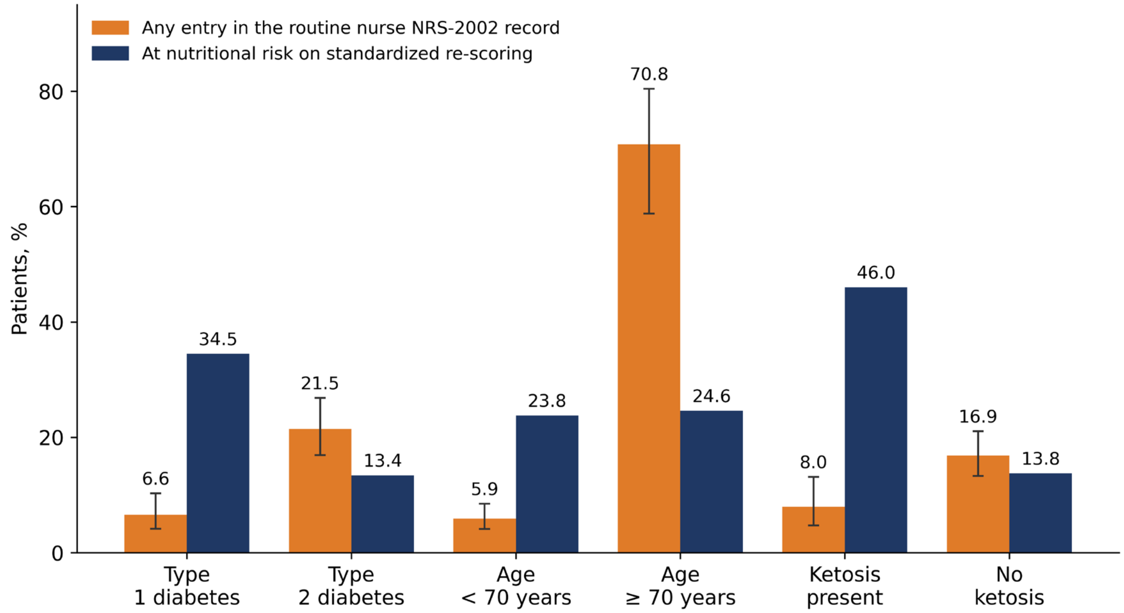

Figure S4. Screening completion (any entry in the routine NRS-2002 record, with 95% Wilson confidence intervals) and nutritional risk detected by standardized re-scoring, across clinical strata.

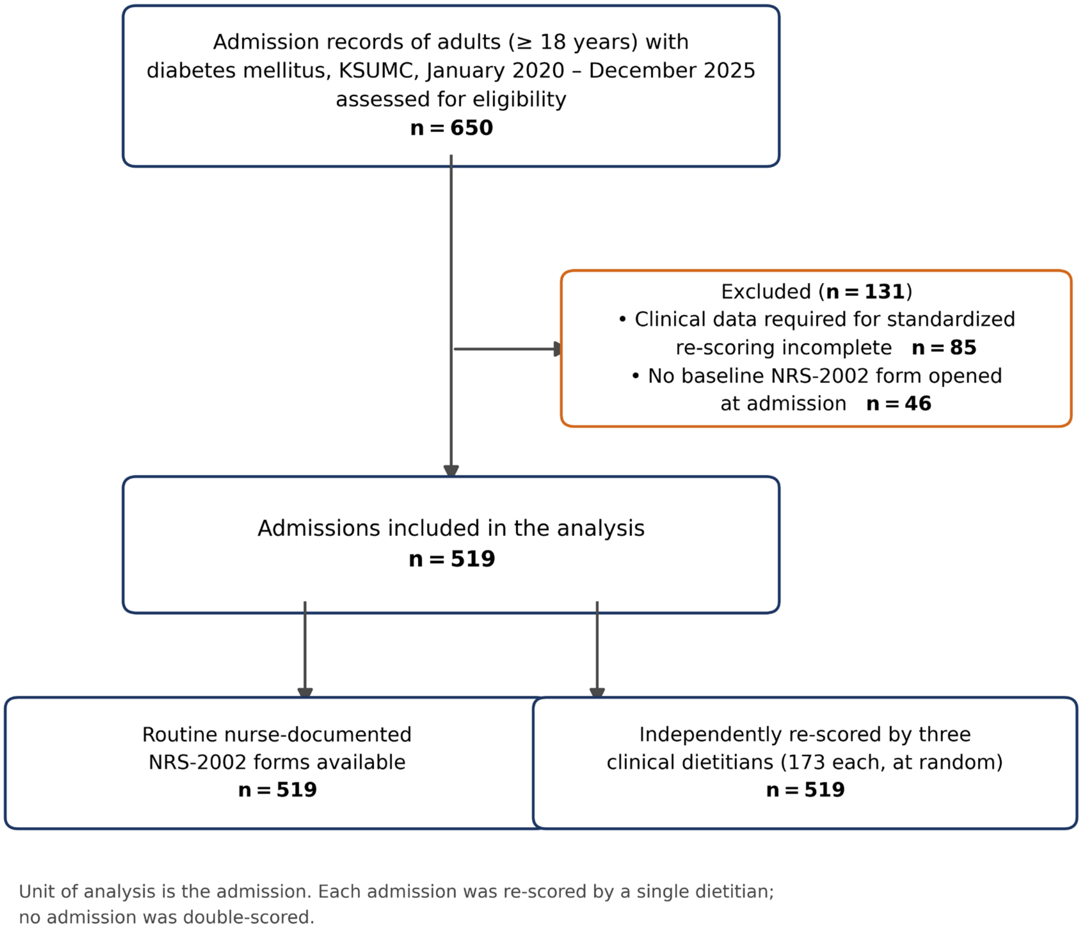

Figure S5. Participant flow. Of 650 admission records of adults with diabetes assessed for eligibility, 131 were excluded — 85 because the clinical data required for standardized re-scoring were incomplete and 46 because no baseline NRS-2002 form had been opened at admission — leaving 519 admissions for analysis. The unit of analysis is the admission. Every included admission had a routine nurse-documented NRS-2002 form and was re-scored by one of three clinical dietitians, the cohort having been divided equally and at random between them (173 each); no admission was double-scored. All admissions of adults with diabetes during the study period were assessed; no sampling was applied. The implementation pathway across all 650 assessed admissions is given in Table 1 of the main text.

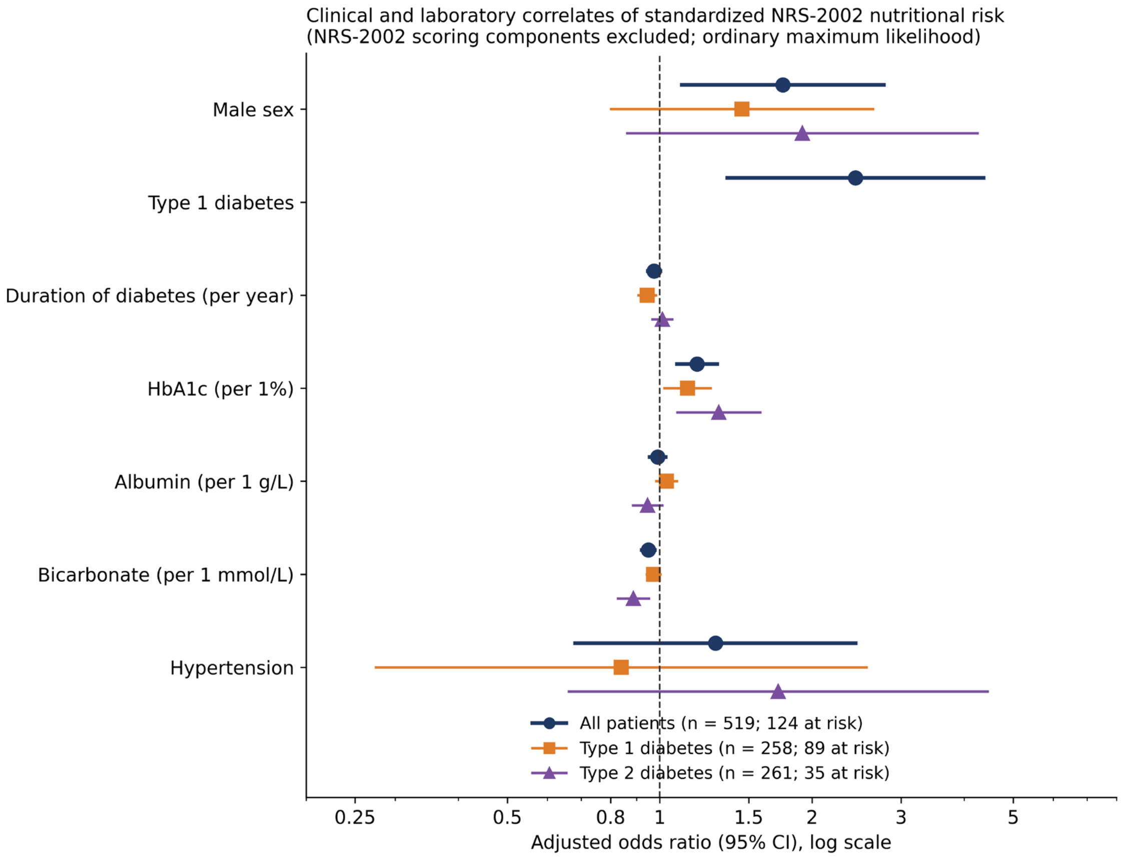

Figure S6. Adjusted associations between non-component clinical and laboratory variables and NRS-2002-defined nutritional risk on standardized re-scoring, in the whole cohort and within diabetes-type strata. NRS-2002 scoring components (body mass index and age) were excluded on structural grounds. All models were fitted by ordinary maximum likelihood; the type 2 diabetes stratum contains 35 events for six terms, so its estimates are unstable and are not interpreted individually.
